# Honey Bee AMPs as a Novel Carrier Protein for the Development of a Subunit Vaccine: An Immunoinformatic Approach

**DOI:** 10.3390/cimb48010081

**Published:** 2026-01-14

**Authors:** Roy Dinata, Piyush Baindara, Chettri Arati, Guruswami Gurusubramanian

**Affiliations:** 1 Department of Zoology, Mizoram University, Aizawl 796004, Mizoram, India; 2 Animal Science Research Center, Division of Animal Sciences, University of Missouri, Columbia, MO 65211, USA; piyush.baindara@gmail.com; 3 National Swine Testing Center, University of Missouri, Columbia, MO 65211, USA

**Keywords:** antimicrobial peptides, honey bee, infectious diseases, immunogenic, carrier-protein, subunit vaccine, immunoinformatics

## Abstract

Infectious diseases remain a persistent global health threat, intensified by the rapid emergence of antibiotic-resistant pathogens. Despite the transformative impact of antibiotics, the escalating resistance crisis underscores the urgent need for alternative therapeutic approaches. Antimicrobial peptides (AMPs) have emerged as promising candidates due to their broad-spectrum antimicrobial and immunomodulatory activities. The present study investigated 82 honey bee antimicrobial peptides (BAMPs) representing seven families: abaecin, apamin, apisimin, apidaecin, defensin, hymenoptaecin, and melittin among eight honey bee species. Immunoinformatics analyses identified five peptides (P15450, A0A2A3EK62, Q86BU7, C7AHW3, and I3RJI9A) with high antigenicity and non-allergenic profiles. Structural modeling, molecular docking with TLR3 and TLR4-MD2, and molecular dynamics simulations revealed stable receptor-peptide interactions and favorable binding energetics, further supported by silico immune simulations. Overall, these findings suggest that the selected BAMPs exhibit strong immunogenic potential and may serve as effective adjuvants or carrier molecules in subunit vaccine design against drug-resistant pathogens; however, further experimental validation is essential to confirm their safety and immunological efficacy.

## 1. Introduction

Among infants and elderly patients, infectious diseases are the primary cause of high mortality. The only defense we have against a wide range of infectious diseases is vaccinations. A very immunogenic carrier protein combined with a highly antigenic but non-immunogenic antigen (haptens) is an effective vaccine development strategy known as the subunit vaccine [1]. AMPs, which are small molecules made up of 5 to 100 amino acids, have been found in a wide variety of organisms, including plants, bacteria, fungi, vertebrates, and invertebrates, including insects [2]. These are cationic molecules with antibacterial, antifungal, antiviral, and antiparasitic properties [3]. With over a million species identified, insects are the most diverse and largest class of living organisms on Earth, due to their capacity for adaptive change and resistance to a broad range of pathogens [4]. Their immune system, which depends completely on innate immunity and provides a highly developed immune response, enables a broad and quick reaction to a variety of microorganisms. Reactive oxygen (ROS) and nitrogen (RNS) species production, the generation of antimicrobial peptides, and the enzymatic cascade that controls hemolymph melanization and coagulation activation are all components of the humoral immune response [5]. Currently, one of the major healthcare challenges is the growing problem of antibiotic resistance [6]. The interest in AMPs has grown over time due to the constant demand to find alternatives to AMPs. Additionally, in the recent past, AMPs have become an attractive point for the scientific community because of their unique antimicrobial properties and mechanism of action to fight against infectious and drug-resistant pathogens [7,8,9]. The first antimicrobial peptides derived from insects were identified and isolated from the lepidopteran *Hyalophora cecropia* by Boman et al. in the 1980s. Several more AMPs have been identified since then. Insects are thought to be one of the most inventive and abundant sources of these molecules because of their high level of biodiversity.

BAMPs are small peptide molecules that are amphipathic, coiled, positively charged, and α-helical. The honey bee has been found to have only 15 AMPs (apidaecin, abaecin, hymenoptaecin, defensin, royalisin, jelleine, apisimin, 10 HDA, apalbumina, apamin, melittin, melectin, adolapin, secapin, and tertiapin) known to date [10]. Four groups of antimicrobial peptides are produced by the hemolymph of bees, which include apidaecins, abaecins, hymenoptaecins, and defensins. These peptides have a broad spectrum of antimicrobial activity, while the royal jelly contains jelleines (IIV), royalisin, HDA, apalbumina, and apisimin (serine-valine-rich peptide) that have antimicrobial, mast cell degranulating, and hemolytic properties. Adolapin, secapin (rich in proline), melectin (rich in lysine), melittin (rich in leucine-alanine), apamin (rich in leucine-cystine), and tertiapin (rich in cysteine-lysine) are among the other bioactive peptides found in bee venom, which may serve as a novel therapeutic target [10].

According to recent research, TLR activation is critical for vaccination memory and herd immunity. Two of the most important TLRs in bacterial infections are TLR-2 and TLR-4. Endotoxins, known as lipopolysaccharides, are selectively recognizable by the TLR4-MD2 complex’s heterodimer receptor [11,12]. With the assistance of CD14 and LPS-binding protein (LBP), lipopolysaccharides are transported to the heterodimer complex [13]. A series of interactions is involved in TLR4’s recognition of Gram-negative bacteria. First, the LBP binds to the LPS and transfers it to CD14. Next, CD14 moves the LPS to the TLR4-MD2 complex. In addition, lipoproteins, lipoteichoic acid (LTA), and lipoglycans can be bound by CD14 [14]. The TLR4-MD2 complex uniquely recognizes a highly conserved domain in LPS that is made up of lengthy acyl chains. TLR4 function was restored by the MD2 complex, as a deletion mutation of TLR4 was unable to recognize LPS. TLR4 and MD2 work together to detect the bacterial lipopolysaccharide (LPS). Particularly, TLR-3 plays a role in viral infections [15]. The endoplasmic reticulum of uninfected cells is where TLR3 is located. The translocation of TLR3 from the endoplasmic reticulum to the cell surface is stimulated by the viral infection. Membrane protein UNC-93B is involved in a pH-dependent dimerization process that activates TLR3. TLR3 primarily identifies 45 bp-long viral dsRNAs; the size of the viral dsRNA is important for signaling [15]. The two components of the immune system are innate and adaptive immunity. Adaptive immunity is highly specific and functions by identifying a foreign agent through T cells using cell-mediated toxicity and complement activation, or B cells using antibodies. Innate immunity involves quick, nonspecific defense mechanisms for antigen clearance. Cytotoxic T Lymphocytes and Helper T Lymphocytes epitope prediction revealed highly antigenic surface epitopes, which were capable of mounting a strong adaptive immune response [16]. Therefore, the presence of B-cell epitope, IFN-γ epitope, and antigenicity is crucial for facilitating a long-lasting memory and herd immunity [17].

Immunoinformatics is the branch of bioinformatics that enables the study of immune system biology through the use of statistical methods, mathematical models, and bioinformatics methods [18]. In a multidisciplinary team, computer scientists and modeling specialists collaborate closely with immunologists to develop immunoinformatics tools, databases, and models. Numerous modeling and computational approaches have been used to address issues in immunology, such as quantifying data produced in lab experiments and deriving biologically significant information regarding their kinetics [18]. In this study, a comprehensive immunoinformatic approach was employed to characterize the immunogenic potential of BAMPs for the first time. Molecular docking analyses were performed to investigate the interactions of BAMPs with Toll-like receptors TLR3 and TLR4-MD2, followed by molecular dynamics simulations using the GROMACS package to evaluate the structural stability and conformational dynamics of the resulting receptor–peptide complexes. The antigenic profiles of the peptides were further assessed through B-cell and T-cell epitope prediction algorithms to identify regions capable of eliciting adaptive immune responses. Finally, in silico immune simulation analyses were conducted to validate the capacity of the top-ranked BAMPs to induce balanced humoral and cellular immune responses.

## 2. Materials and Methods

### 2.1. Amino Acid Sequence Retrieval of BAMPs

**Figure 1:**
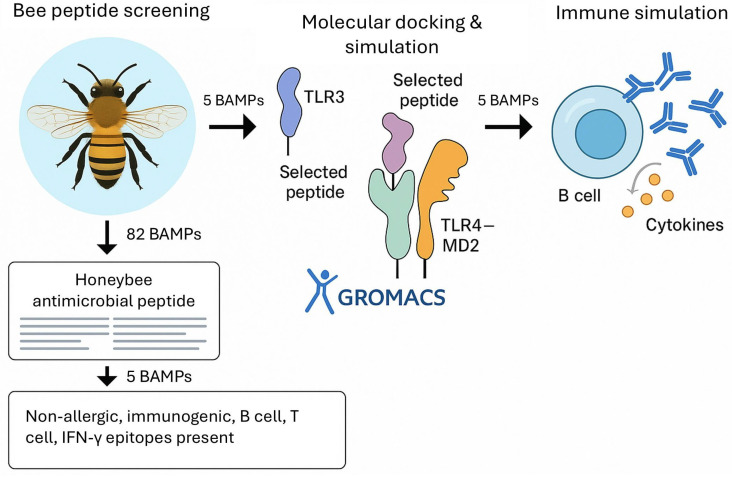
General workflow and overview of the current study.

The total 82 BAMP sequences belonging to seven groups of AMPs (Abaecin: 10, Apamin: 4, Apisimin: 4, Apidaecin:23, Defensin:24, Hymenoptaecin:10, Melittin: 7) from seven honey bee species (*Apis mellifera*, *A. cerana cerana*, *A. cerana japonica*, *Melipona quadrifasciata*, *A. mellifera carnica*, *A. dorsata*, and *A. andreniformis*) are obtained from the Uniprot database (https://www.uniprot.org (accessed on 6 October 2024)) in FASTA format (Table S1). Retrieved BAMPs are further used as ligand molecules to check their immunogenic properties. The overall experimental design is represented in Figure 1.

### 2.2. Homology Modeling and 3D Structure Prediction of BAMPs

After retrieving the BAMP sequences, the SWISS-MODEL database was used to obtain the three-dimensional structure of BAMPs [19]. SWISS-MODEL used two database search methods: BLAST (version 2.14.1), which is fast and sufficiently accurate for closely related templates, and HHblits, which adds sensitivity in case of remote homology [20,21]. Next, the BAMP templates are ranked according to the expected quality of the resulting models, as estimated by the Global Model Quality Estimate (GMQE) and Quaternary Structure Quality Estimate (QSQE) [22,23]. Top-ranked templates are then selected to build the final model. After that, the 3D structures of BAMPs are saved in PDB format for additional study (Figure S1).

### 2.3. Validation of 3D Structure

Using SAVES v6.0 (https://saves.mbi.ucla.edu (accessed on 6 October 2024)), a comprehensive toolkit package of five tools: ERRAT, VERIFY3D, PROVE, PROCHECK, and WHAT CHECK, that validated various types of stereochemical properties of the protein structure. On the other hand, PROCHECK is used for the tertiary structure validation of the homology-modeled AMPs. The PROCHECK analyses the overall model geometry using residue-by-residue geometry and provides the stereochemical quality (91.4% (470), 6.8% (35), 1.0% (5), and 0.8% (4) residues belong to the most favored regions, additionally allowed regions, generously allowed regions, and disallowed regions, respectively) of a predicted model [24]. Overall, a good quality 3D structure model is considered to have over 90% in the most favored regions. Further three-dimensional structure validation was performed using PROSA (https://prosa.services.came.sbg.ac.at (accessed on 6 October 2024)) Z-score. Protein Structure Analysis, or ProSA, is a well-known program with a significant user base that is widely used for modeling and structure prediction as well as for optimizing and validating empirical protein structures [25] (Figure S1).

### 2.4. Retrieval of Receptor Protein and Structure Preparation

Crystal structures of human TLR3 (PDBID: 1ZIW) and TLR4-myeloid differentiation factor 2 (MD2) (PDBID: 3FXI) are retrieved from RCSB Protein Data Bank (https://www.rcsb.org (accessed on 6 October 2024)). Water molecules, metal ions, and HET atoms from the receptor structures are removed before docking using the BIOVIA Discovery Studio Visualizer (https://discover.3ds.com/discovery-studio-visualizer-download (accessed on 6 October 2024)) and PyMol (version 2.6.2) [26,27]. Additionally, polar hydrogen atoms and partial charges are added to the receptors using UCSF Chimaera 1.15 (https://www.cgl.ucsf.edu/chimera (accessed on 6 October 2024)) [28]. Using the built-in dock prep program in the Chimaera tool, truncated side chains in the receptor are modified, along with the inclusion of Gasteiger charges in the target receptor proteins. For method validation and docking, the target receptor protein preparation findings were saved in a PDB file format (Figure S2).

### 2.5. Helper T Lymphocytes (HTL) Epitope Prediction

The IEDB server (http://tools.iedb.org/mhcii/ (accessed on 6 October 2024) is used to predict the Helper T Lymphocytes (HTL) epitope of BAMPs [29]. Based on IC_50_ values and percentile rank, all of the epitopes for mouse MHC class II alleles (IAb, IAd, IAs, IEb, IEd, and IEs) are predicted [30]. The IC_50_ values of the peptides with the highest affinity are 50 nm, those with intermediate affinity are 500 nm, and those with the lowest affinity are 5000 nm. By comparing peptide IC_50_ values to a set of randomly selected antigens taken from the SWISS-PROT database, a percentile rank is produced. Overall, the most affinity-predicted epitopes were those with the lowest IC_50_ value.

### 2.6. Cytotoxic T Lymphocyte (CTL) Epitope Prediction

The development of subunit vaccines depends on the prediction of CTL epitopes that are essential to elicit the desired immune response. Based on MHC class-I binding ability, TAP transport efficiency, and proteasomal C-terminal cleavage, NetCTL 1.2 is used to predict the CTL epitopes for BAMPs [16]. The scores of all three predictions were combined, and the threshold for CTL epitope identification was set at 0.75.

### 2.7. B-Cell Epitope Prediction

BCPRED: The B cell epitope prediction Server is used for the prediction of linear B-cell epitopes for the BAMPs (http://ailab.ist.psu.edu/bcpred/predict.html, accessed on 6 October 2024)), which is collectively based on AAP, BCPred, and FBCpred [30]. The AAP approach is based on the finding that some specific amino acid pairs appear more frequently in epitope than non-epitope sequences [17].

### 2.8. Interferon-Gamma (IFN-γ) Epitope Prediction

IFN-γ plays a significant role in both the innate and adaptive immune response. The IFN-γ epitope server (https://webs.iiitd.edu.in/raghava/ifnepitope/predict.php, accessed on 6 October 2024)) is used to predict the IFN-γ epitopes [31]. The IFN-γ epitope server is built on a dataset of IFN-γ-inducing and non-inducing MHC class-II binders that can activate T-helper cells, whereas a hybrid that employs a support vector machine (SVM) and motif is used for the prediction [32].

### 2.9. Allergenicity and Antigenicity Prediction

Allergenicity and immunogenicity prediction are an essential step to further development of BAMPs in biopharmaceuticals. The allergenicity of BAMPs is examined using the Allerdictor rapid allergen prediction program (http://allerdictor.vbi.vt.edu, accessed on 6 October 2024), where the prediction of the allergens and non-allergens is based on a traditional SVM method [33]. To determine the antigenicity of the BAMPs, the ANTIGENpro web server (http://www.scratch.proteomics.ics.uci.edu, accessed on 6 October 2024) is employed, where the prediction is based only on the input sequence, independent of all alignments and pathogen identities. The two-step prediction process used by ANTIGENpro is based on four algorithms and several sequence representations [34]. Finally, the SVM classifier produced a final summary prediction result that indicates the likelihood that a peptide would possess antigen-specific features. The antigen prediction tool VaxiJen v2.0 (http://www.ddg-pharmfac.net/vaxijen/VaxiJen/VaxiJen, accessed on 6 October 2024) was used to assess. BAMPs for further confirmation of antigenicity. To provide optimal accuracy, a cutoff value of 0.5 was employed to assess the antigenicity for all BAMPs [35].

### 2.10. Molecular Docking

The PatchDock algorithm, which is based on shape complementarity criteria including molecular shape representation, surface patch matching, filtering, and scoring, is used to accomplish molecular docking. Moreover, the FireDock algorithm (Fast Interaction REfinement in molecular DOCKing) at https://bioinfo3d.cs.tau.ac.il/FireDock, accessed on 6 October 2024, optimizes the docked structures. The complex type is set to “default,” and the clustering RMSD was adjusted to 1.5 for protein-protein molecule docking. The first step of the PatchDock approach is a rough refinement using RISCO (restricted interface side-chain optimization) with atomic radii (0.8 scales) to account for steric conflicts. The rigid-body optimization (RBO) method scores and ranks the updated docked complexes based on an energy function and then generates an output. The PatchDock server provides the docked complexes (100 top-hits) for every protein receptor, together with information on their scores, areas, ACEs (atomic contact energies), and transformation scores [36,37]. Subsequently, possible receptor–ligand interactions are analyzed using a Discovery Studio visualizer. The top ten hits of docked complexes are then used to rerun the FireDock analysis through the PatchDock web server’s “refine best solutions with FireDock” feature, allowing for final refining. In this iterative run, every docked complex generated by the PatchDock analysis is optimized using full interface side-chain optimization (FISCO, atomic radii: 0.85 scale). Selecting the movable residues, calculating the rotamer energy for side-chain flexibility, building the side-chain optimization problem solved by integer linear programming, and refining the relative positions of the docking partners using Monte Carlo minimization to obtain the binding score function are some of the steps involved in the FISCO refinement process [38]. Based on the binding score, ACE, partial electrostatics, binding-free energy estimates, and attractive and repulsive van der Waals interactions, the improved docked complexes of receptors and ligands are ranked [39,40,41].

### 2.11. Molecular Dynamics and Simulation

To perform the molecular dynamics simulation studies, the top hit (pose1) receptor–ligand docked complexes of TLR3_BAMPs (TLR3_Abaecin, TLR3_Apamin, TLR3_Apisimin, TLR3_Hymenoptaecin, and TLR3_Melittin) and TLR4-MD2_AMPs (TLR4_Abaecin, TLR4_Apamin, TLR4_Apisimin, TLR4_Hymenoptaecin, and TLR4_Melittin) are selected. The GROMACS (GROningen MAchine for Chemical Simulations, v5.1.5) program is used to perform the MD Simulation [42,43]. The following steps are sequentially included in the process of MD simulation: (i) Generation of receptor and ligand topology and conversion of the receptor and ligand PDB file format to gmx format (pdb2gmx). To generate the force field and parameter files for the TLR receptors and BAMPs ligands, the GROMOS96 43A1 force field and acpype (a Python (3.10) interface to Antechamber writing GROMACS topologies) are used, respectively [38]; (ii) Specification of a grid box (cubic, 10), and solvation (a three-point water model, TIP3P); (iii) Addition of ions (to neutralize the system, Na^+^/Cl^−^ ions are introduced as counter ions); (iv) Next, to remove steric conflicts (atomic location) and structural defects in the bond length and bond angle, energy minimization (1000 steps and 10 kJ/mol/nm tolerance) is carried out; (v) Equilibration consists of two stages, first, phase-I that apply restrictions to the ligand, that contained constant number of atoms, volume, and temperature (NVT). Using a Berendsen thermostat coupling, the entire ensemble equilibration is performed for 500 ps at 300 K. The second step, phase-II, has a constant number of atoms, pressure, and temperature (NPT) and is where temperature coupling groups are treated state that the total ensemble equilibration is then performed for 1000 ps; (vi) The Particle Mesh Ewald (PME) approximation, which computes long-range electrostatic interactions by setting a cut-off radius of 10 Å, is included in the MD generation process [21]. The Linear Constraint Solver (LINCS) algorithm limits bond lengths. The unrestrained production of MD simulations was conducted at 100 ns, 300 K temperature using the Langevin coupling technique, and 1 bar pressure using the Nose I-Hoover Langevin Piston algorithm to obtain data. Every simulation was run for 100 ns at a 4-fs time step, and after 50 ps, each trajectory was kept. Before performing additional analysis, the system is equipped to its start position based on the backbone of the receptor, and the receptor–ligand complex trajectory is corrected using periodic boundary conditions (PBCs). PyMol and VMD are visualization tools that are used to display the simulated results [27,44]. Subsequently, simulation snaps are utilized to compute the solvent-accessible surface area (SASA), the number of hydrogen bonds between the ligand and the receptor, the radius of gyration (Rg), the root-mean-square deviation (RMSD), and the root-mean-square fluctuation (RMSF) values within the GROMACS utilities [45,46].

### 2.12. Binding-Free Energy Calculation (MM-PBSA)

Using molecular mechanics potential energy (electrostatic and van der Waals interactions) and solvation-free energy (polar and non-polar solvation energies) protocols, which are carried out by the g_mmpbsa suite of Molecular Mechanics Poisson–Boltzmann Surface Area (MM-PBSA), the binding-free energy of the TLRs receptor–ligand complexes obtained from FireDock is calculated [10]. The free energy can be calculated using the following formula: GX (receptor–ligand complex) = EMM (MM potential energy in vacuum) + GP (polar solvation energy) + GNP (non-polar solvation energy). Trajectory profiles are preserved every 50 ps, and MM-PBSA is computed using a short trajectory of the last 80 ns from the entire MD trajectory (100 ns). Next, an SASA model is applied to determine the non-polar solvation energy. The influence of each residue’s binding energy contribution on the total binding energy is examined using the Python tool ‘MmPbSaDecomp.py’ (g_mmpbsa suite, version 1.6.1) [45].

### 2.13. Principal Component Analysis (PCA)

To identify the physiologically relevant protein domains shifting from the improperly limited motions of the atoms, PCA modifies the complexity of the data available on TLR receptor–ligand complexes. Through the use of uncorrelated variables, the main components transform correlated data into uncorrelated variables that may be used to extrapolate variance. A covariance matrix is generated in PCA by dividing the translational and rotational movements of the protein and atoms from the molecular dynamics simulation system using the gmx covar from GROMACS. Correlated motion between the two Cα atoms is represented by positive values for the covariance matrix, whereas anti-correlated motion is represented by negative values. After the original trajectory was filtered, the most significant eigenvectors, 1 and 2, were computed using the gmx analysis from the GROMACS utilities [47]. Lastly, the gmx anaeig tool and PAST (version 1.86b) software are used to find the overlap between the computed PCs and the trajectory’s coordinates [48].

### 2.14. Immune Simulations

The C-ImmSim server is used to run in silico immune simulations for each of the BAMPs to investigate the unique immunogenicity of each B AMP independently [49]. This server simulates three distinct compartments for immune cells in mammals: the bone marrow, the thymus, and a tertiary lymphatic organ like the lymph node. It then uses a machine learning algorithm and a position-specific scoring matrix (PSSM) to predict the immune epitopes and immune interaction [50]. Three in silico injections with time steps of 1, 84, and 170 are administered at 4-week intervals to carry out the immunological simulation. Time step 1 is regarded as the initial injection at the 0-time point, and the one-time step is equal to an eight-hour day. A total of 1050 simulation steps is used, which is roughly equal to a year and a half [51]. The C-ImmSim server’s default values are maintained for all other settings.

## 3. Results

### 3.1. Tertiary Structure Modeling, Refinement, and Validation of BAMPs

Among the 82 predicted homology modeled BAMPs structure, Ramachandran plot validation recommended 17 structures of BAMPs [5 Abaecin (AOA2A3E7A0, B9UK30, B9UK35, B9UK38, and P15450), 4 Apamin (A0A2A3EK62, B7UUK0, P01500, and Q86QT2), 3 Apisimin (A0A2A3EKN9, Q8ISL8, and Q86BU7), 1 Hymenoptaecin (C7AHW3), and 4 Melittin (I3RJI9, P0DPR9, P01501, and P68407)] based on the favored regions score > 90% (Table S2). Further validation of tertiary structure was performed based on the PROSA z-score of BAMPs, score ≤ −6.0 are considered as stable structures. BAMPs z-score for Abaecin (P15450): −3.16, Apamin (A0A2A3EK62): −1.1, Apisimin (Q86BU7): −4.98, Hymenoptaecin (C7AHW3): −3.39, and Melittin (I3RJI9A): −2.61, which signifies the stability of five structures out of 17 BAMPs (Figure S1).

### 3.2. CTL, HTL, B-Cell, and Interferon γ Epitopes of BAMPs

Based on the result of tertiary structure prediction and validation, five BAMPs were selected for downstream analysis as our work pipeline needs a three-dimensional structure for further molecular docking simulation study. Furthermore, the five BAMPs (Abaecin: P15450, Apamin: A0A2A3EK62, Apisimin: Q86BU7, Hymenoptaecin: C7AHW3, Melittin: I3RJI9A) with highly immunogenic and non-allergenic properties were subjected to immunoinformatics analysis. Based on the results of the Cytotoxic T Lymphocyte (CTL) epitope prediction, BAMPs exhibited three to seven CTL epitopes (abacein-3, apisimin-5, hymenoptaecin-3, and melittin-7), with a combined score ranging from 1.3281 to 2.9246 (Table 1); however, CTL epitopes were not found in apamin. Low percentile ranks of anticipated HTL epitopes that can effectively elicit a helper T cell response further reflect the strong antigenic qualities. All five BAMPs possessed HTL epitopes. A higher number of HTL epitopes were observed in hymenoptacein (3), followed by apisimin (2), melittin (2), apamin (1), and abaecin (1), with percentile ranks ranging between 0.22 and 2.10 (Table 2). Except for abaecin, all four BAMPs possessed B-cell epitopes. The highest number of B-cell epitopes was observed in hymenoptaecin (16), followed by apisimin (10), apamin (1), and melittin (3). All B-cell epitopes exhibited scores ranging between 0.83 and 1.0, which were within the threshold scores ≥ 0.70 (Table 3). Two of the BAMPs, hymenoptaecin and melittin, exhibited IFN epitopes among the five. Hymenoptaecin exhibited two IFN epitopes with scores ranging from 0.47729732 to 0.47729732, while melittin possessed a single IFN epitope with a score of 0.3526523. Eleven IFN epitopes were found in apidaecin, with a cumulative score ranging from 0.38320905 to 0.85811575 (Table 4).

### 3.3. Selected BAMPs Are Antigenic but Non-Allergenic

VaxiJen score five BAMPs suggested the presence of antigenic properties: abaecin (P15450): 0.6327, apamin (A0A2A3EK62): 0.3800, apisimin (Q86BU7): 0.4738, hymenoptaecin (C7AHW3): 0.8128, and melittin (I3RJI9A): 0.6664, ranged between 0.5736 and 0.7987. Further validation of antigenicity was performed using the ANTIGENpro score, which varied from 0.165312 to 0.595802, confirming the antigenicity of the five BAMPs further and indicating that they were all extremely antigenic (Table 5). Moreover, the AllergenFP Tanimoto similarity index score ≤ 0.9 suggests non-allergenic properties of BAMPs. Out of five BAMPs, melittin (C7AHW3) was characterized as an allergen based on the score 1.0, while hymenoptaecin (C7AHW3): 0.78, apamin (A0A2A3EK62): 0.77, abaecin (P15450): 0.81, and apisimin (Q86BU7): 0.85 were non-allergic (Table 5).

### 3.4. Molecular Docking Revealed High Binding Affinities of BAMPs with TLR3 and TLR4-MD2 Complex

**Figure 2:**
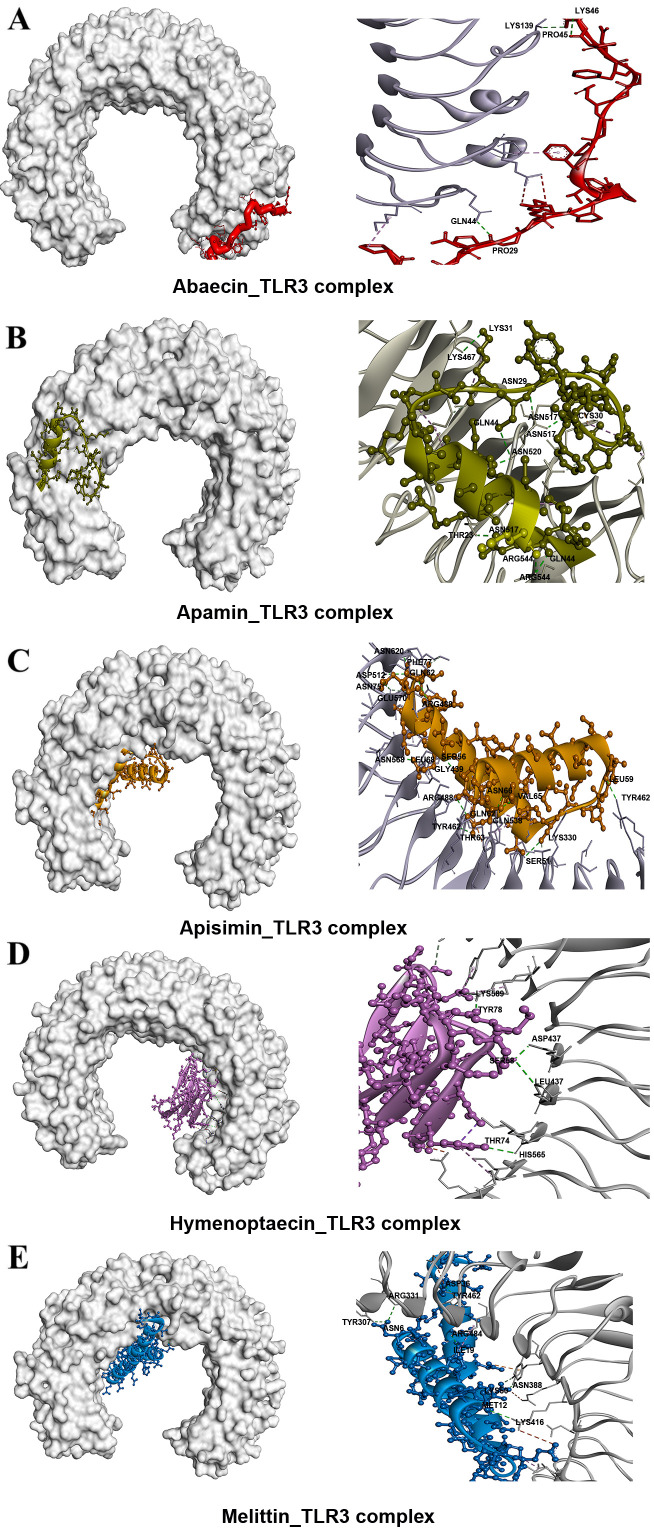
Docked complexes and molecular interactions of selected BAMPs with Toll-like receptor 3 (TLR3): (**A**) Abaecin, (**B**) Apamin, (**C**) Apisimin, (**D**) Hymenoptaecin, (**E**) Melittin.

**Figure 3:**
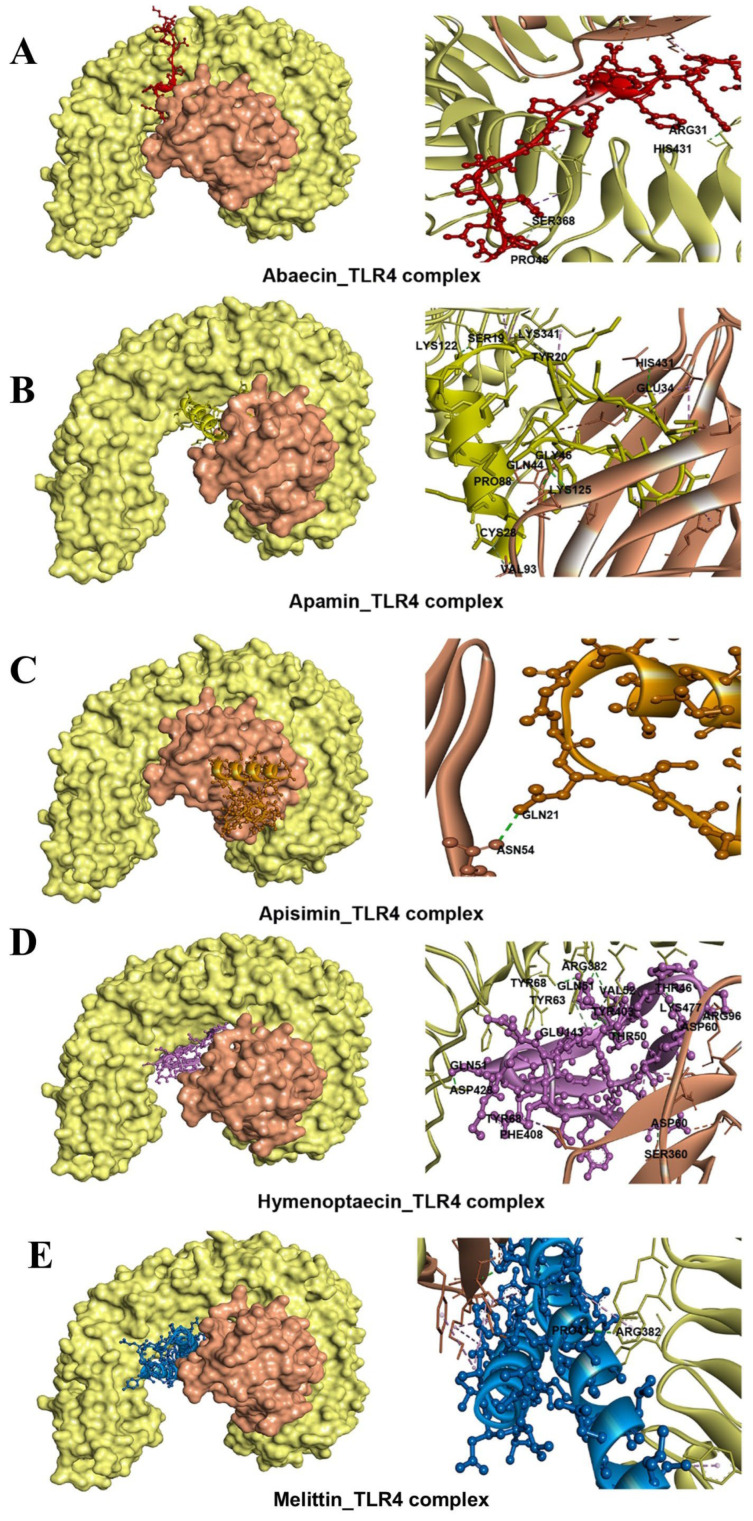
Docked complexes and molecular interactions of selected BAMPs with Toll-like receptor 4 (TLR4): (**A**) Abaecin, (**B**) Apamin, (**C**) Apisimin, (**D**) Hymenoptaecin, (**E**) Melittin.

All the selected 3D high-quality five BAMPs structures were subjected to molecular docking analysis using the Patch Dock server and further refined using the FireDock web server. According to the results of the FireDock tool, abaecin, apisimin, apidaecin, hymenoptaecin, and melittin displayed strong binding affinities with TLR3 and TLR4-MD2 complex. The binding affinity of these top-hit ligands varied from 30.11 to −55.36 kcal/mol and from −16.52 to −50.31 kcal/mol in the case of TLR3 and TLR4-MD2 complex, respectively (Table 6 and Figure 2 and Figure 3). The TLR3_Hymenoptaecin (−55.36) complex showed the highest binding affinities, followed by apamin (−35.23), melittin (−31.41), apisimin (−30.77), and Abaecin (−30.11). Furthermore, the TLR4_Hymenoptaecin (−50.31) complex showed the highest binding affinities, followed by apamin (−34.11), melittin (−23.84), abaecin (−22.51), and apisimin (−16.52) (Tables S3 and S4).

### 3.5. Hydrogen Bonds, Electrostatic, and Hydrophobic Interactions Confirmed the Strong Interaction of BAMPs with TLRs

Apisimin formed four hydrogen bonds (GLN208-ASN75, TYR283-ALA57, VAL39-HIS156, ALA61-TYR283) and nine hydrophobic (3 Alkyl and 6 π-alkyl) bonds with the TLR3 receptor (Table 6 and Figure 2 and Figure 3). Melittin shared seven hydrogen bonds (LEU4-GLU460, ARG331-GLU34, LYS589-LEU56, SER282-ALA21, ASN328-SER17, HIS565-LEU52, THR53-GLU533), three electrostatic bonds (Pi-Anion: 3), and four hydrophobic (Alkyl: 4) bonds with the TLR3 receptor (Table 6 and Figure 2 and Figure 3). Next, abaecin formed three hydrogen bonds (ILE661-PHE37, ASN659-PHE43, TRP660-PHE34) and four hydrophobic (1 Pi-Pi Stacked, 1 Alkyl, and 2 pi-Alkyl) bonds with the TLR3 receptor. Further, hymenoptaecin formed twelve hydrogen bonds (ARG489-ASP69, ASN517-GLY72, SER571-ASN76, SER571-ALA77, GLY573-TYR63, TYR63-LEU595, THR74-ASN515, GLY75-ASN517, ASN596-TYR63, PRO646-GLY80, GLY80-PHE644, ALA519-LEU59), one electrostatic (Attractive Charge), and one hydrophobic (Alkyl) bond with the TLR3 receptor (Table 6 and Figure 2 and Figure 3). Similarly, apamin formed nine hydrogen bonds (ASN517-ASN29, ASN520-GLN44, ARG544-GLN44, ARG544-GLN43, THR23-ASN517, CYS30-ASN517, LYS31-LYS467, LYS493-LYS31, PRO24-ASN541) and seven hydrophobic (5 Alkyl and 2 π-alkyl) bonds with the TLR3 receptor (Table 6 and Figure 2 and Figure 3). On the other hand, TLR4-MD2_ Apisimin formed three hydrogen bonds (ASN54-GLN21, SER360-ALA75, SER472-VAL39) with the TLR4-MD2 receptor. TLR4-MD2_Abaecin docked complex formed three hydrogen bonds (ARG31-HIS431, SER368-PRO45, LYS122-PHE37), one electrostatic (attractive charge), and three hydrophobic bonds (1 Pi-Sigma, 1 Amide-Pi Stackedand 1 Alkyl) with TLR4-MD2 (Table 6 and Figure 2 and Figure 3). Similarly, apamin formed seven hydrogen bonds (HIS431-GLU34, LYS122-SER19, SER19-LYS122, CYS28-VAL93, LYS341-TYR20, PRO88-GLN44, GLY46-LYS125), one electrostatic (attractive charge), and ten hydrophobic (5 Alkyl and 5 pi-alkyl) bonds with the TLR4-MD2 receptor (Table 6 and Figure 2 and Figure 3). Next, hymenoptaecin formed ten hydrogen bonds (SER360-ASP60, ARG382-GLN51, ARG382-GLN51, ARG382-VAL52, ARG382-TYR68, TYR403-THR50, LYS477-THR46, GLN51-ASP428, TYR63-GLU143, ARG96-ASP60), two electrostatic (attractive charge), and ten hydrophobic (4 Alkyl) with TLR4-MD2 receptor. Further, the TLR4-MD2_Melittin docked complex formed three hydrogen bonds (SER472-GLU26, SER472-GLU26, HIS431-LEU4), two electrostatic (1 attractive charge and 1 Pi-Anion), and one hydrophobic (1 Alkyl) bond (Table 6, Tables S3 and S4 and Figure 2 and Figure 3).

### 3.6. Validation of BAMPs-TLRs Interactions by MD Simulation and MM-PBSA Energy Calculations

**Figure 4:**
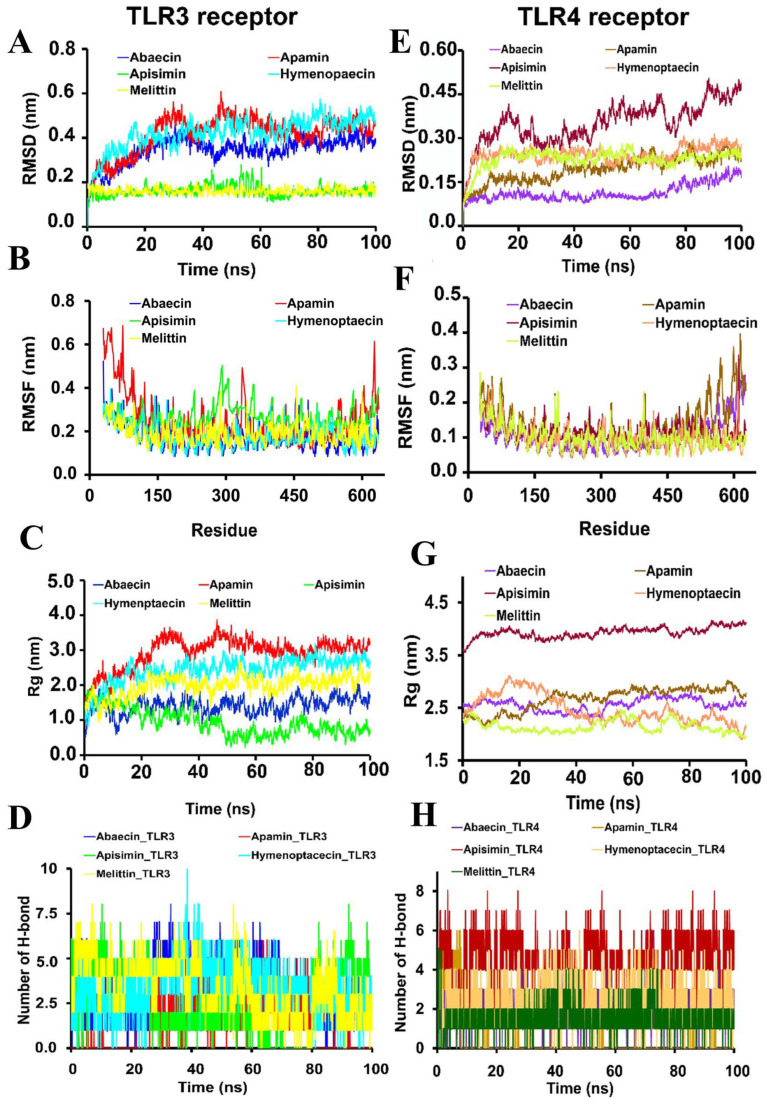
Molecular dynamics simulation analysis of docked complexes between BAMPs and TLR3 and TLR4–MD2 over a 100 ns trajectory. (**A**,**E**) Root-mean-square Deviation (RMSD) showing structural stability, (**B**,**F**) Root-mean-square Fluctuation (RMSF) representing residue flexibility, (**C**,**G**) Radius of Gyration (Rg) indicating compactness, and (**D**,**H**) average number of hydrogen bonds formed during the simulation.

To verify the stability of the docked complexes of receptor ligands under varied thermobaric events, molecular dynamics and simulation investigations were conducted. At constant density, pressure, temperature, and volume, the stability, flexibility, and compactness of the docked complexes of BAMPs and TLR3/TLR4-MD2 were examined. Rg was obtained to determine the complex’s compactness, while RMSD, RMSF, and the number of hydrogen bonds were accessed to determine the complex’s stability and flexibility (Table 6 and Figure 4).

### 3.7. BAMPs-TLRs Docked Complexes Are Energetically Stable During MD Simulations

RMSD of protein backbone for TLR3_BAMPs docked complexes ranged between 0.194 and 0.407 nm (Abaecin, 0.294 nm; TLR3_Apamin, 0.309 nm; TLR3_Apisimin, 0.196 nm; TLR3_Hymenoptaecin, 0.407 nm; and TLR3_Melittin, 0.194 nm). The average RMSD for the TLR4-MD2 receptor backbone was observed as 0.127 for TLR4-MD2_Abaecin, 0.247 for TLR4-MD2_Apamin, 0.175 for TLR4-MD2_Apisimin, 0.38 for TLR4-MD2_Hymenoptaecin, and 0.27 nm for TLR4-MD2_Melittin (Table 6 and Figure 4). Also, low RMSF values are observed within a range from 0.123 to 0.263 nm for all the TLR3 and TLR4-MD2_BAMP docked complexes (TLR3_Abaecin, 0.150 nm, and TLR4-MD2_Abaecin, 0.195 nm; TLR3_Apamin, 0.173 nm, and TLR4-MD2_Apamin, 0.263 nm; TLR3_Apisimin, 0.150 nm, and TLR4-MD2_Apisimin, 0.216 nm; TLR3_Hymenoptaecin 0.097 nm, and TLR4-MD2_Hymenoptaecin, 0.169 nm; TLR3_Melittin 0.123 nm, and TLR4-MD2_Melittin 0.189 nm), which allowed less variation in the flexibility, compactness, and stability of the amino acid residues in the TLR3 and TLR4-MD2 receptors (Table 6 and Figure 4). Furthermore, lower Rg values were also observed for the TLR3/TLR4-MD2_*B* AMP complexes (TLR3_Abaecin: 1.701, TLR4-MD2_Abaecin: 2.556, TLR3_Apamin: 3.187, TLR4-MD2_Apamin: 2.968, TLR3_Apisimin: 1.687, TLR4-MD2_Apisimin: 3.931, TLR3_Hymenoptaecin: 2.486, TLR4-MD2_Hymenoptaecin: 2.167, TLR3_Melittin: 2.399, TLR4-MD2_Melittin: 2.197). A lower Rg number suggested more compactness, correct protein folding, and a more stable structure of the receptor–ligand complex (Table 6 and Figure 4). Further, the stability of the receptor–ligand complex was determined by the number of H-bonds formed between them. A higher number of hydrogen bonds maintained throughout the simulation represents a more stable complex. The average higher number of H-bonds maintained in the TLR3_ Hymenoptaecin (3.32249) followed by Melittin_TLR3 (3.162048), Abaecin_TLR3 (3.063052), Apisimin_TLR3 (2.409237), and Apamin_TLR3 (1.407631) complexes during the 100 ns simulation experiment. For TLR4-MD2 receptors, the highest average number of H-bonds was observed in the Apisimin_TLR4-MD2 (4.413323), followed by Melittin_TLR4-MD2 (4.399389), Hymenoptaecin_TLR4-MD2 (2.263242), Abaecin_TLR4-MD2 (1.971108), and Apamin_TLR4-MD2 (0.353933) complexes during the 100 ns simulation experiment (Table 6 and Figure 4).

### 3.8. Molecular Mechanics Poisson-Boltzmann Surface Area Binding Energy Analysis for BAMPs-TLRs Complexes

The binding free energy of abaecin (TLR3 −71.935, TLR4 −67.206), apamin (TLR3 −158.878, TLR4 −70.830), apisimin (TLR3 −63.034, TLR4 −77.920), hymenoptaecin (TLR3 −109.491, TLR4 −109.122), and melittin (TLR3 −116.62, TLR4 −117.133) were observed between −63.034 to −117.133 kcal/mol (Table 6). Further, van der Waals energy (EvdW, kJ/mol), electrostatic energy (Eelec, kJ/mol), polar solvation energy (Epolar, kJ/mol), solvent accessible surface area (SASA energy, Enonpolar, kJ/mol) were calculated for TLR-BAMP complexes (Table 6). Residual decomposition analysis showed that the amino acid residues of the TLRs receptors contributed to better binding to the BAMPs (Figure 4).

### 3.9. Principal Component Analysis for BAMP-TLR Complexes

**Figure 5:**
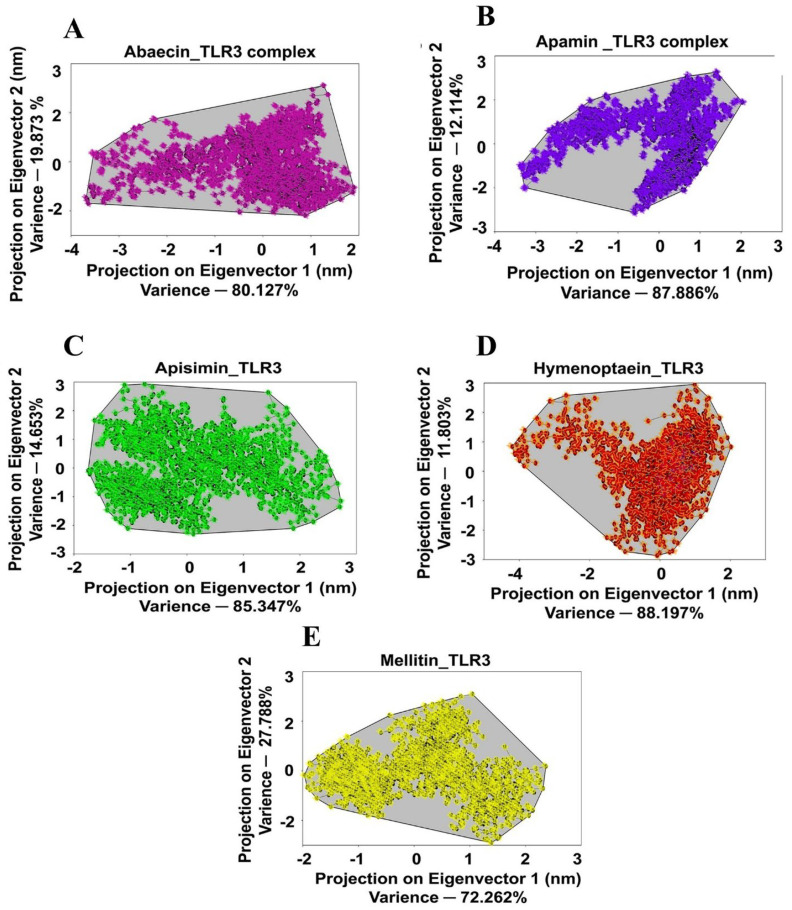
PCA scatter plots showing the projections of the displacement of C_α_ atoms along the first eigenvector and the second eigenvector at each time point for the BAMPs complexes with TLR3. (**A**) Abaecin, (**B**), Apamin, (**C**) Apisimin, (**D**) Hymenoptaecin, (**E**) Melittin.

**Figure 6:**
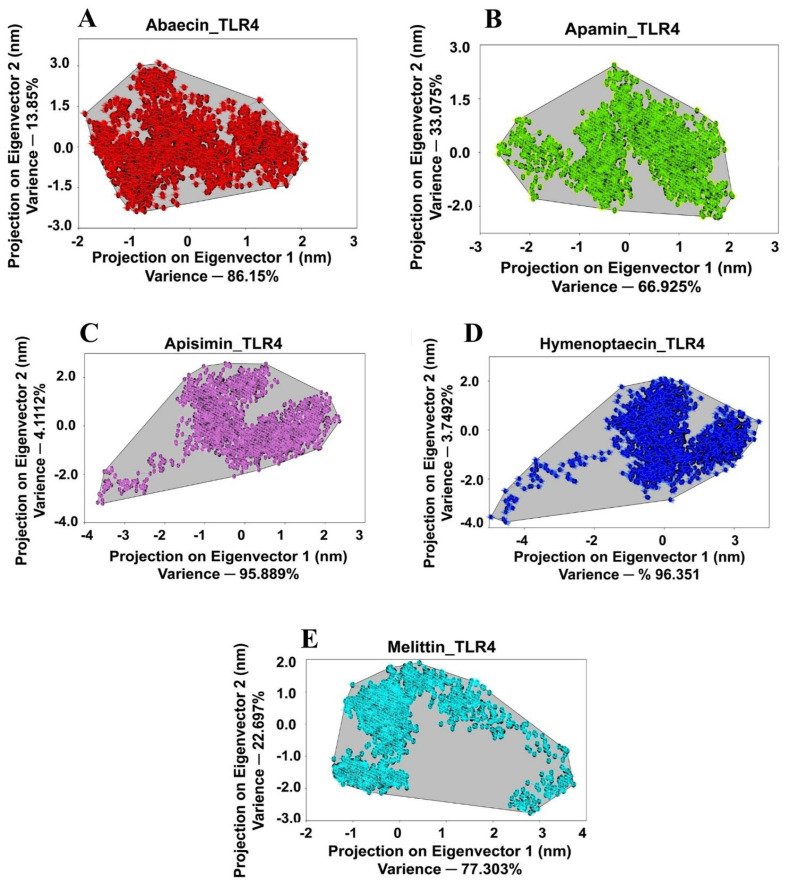
PCA scatter plots showing the projections of the displacement of C_α_ atoms along the first eigenvector and the second eigenvector at each time point for the BAMPs complexes with TLR4-MD2. (**A**) Abaecin, (**B**) Apamin (**C**) Apisimin (**D**) Hymenoptaecin (**E**) Melittin.

Next, based on the relative motion of Cα atoms in simulation trajectories, PCA was used to evaluate the stability of the receptor and ligand complexes. Clusters created by the projection of eigenvectors 1 and 2, respectively, represented the total movement of Cα atoms in the complex systems of the receptor protein. While a positive score for eigenvectors 1 and 2 suggests that the Cα atoms are moving in the same and equal directions, a negative score in the MD simulation trajectories suggests that the Cα atoms are going in the opposite direction. In comparison to an unstable cluster, an exceptionally stable cluster takes up less phase space. Two clusters are more distinctly defined and occupy less phase space in the 2D projections of the TLR3/TLR4-MD2_BAMP complexes. The following was noted regarding PC1 and PC2’s percentage variance. PC1: TLR3_Abaecin: 80.127, TLR4-MD2_Abaecin: 86.150, TLR3_Apamin: 87.886, TLR4-MD2_Apamin: 66.925, TLR3_ Apisimin: 85.347, TLR4-MD2_Apisimin: 95.889, TLR3_Hymenoptaecin: 88.197, TLR4-MD2_Hymenoptaecin: 96.351, TLR3_Melittin: 72.262, TLR4-MD2_Melittin: 77.303, and PC2: TLR3_Abaecin: 19.873, TLR4-MD2_Abaecin: 13.850, TLR3_Apamin: 12.114, TLR4-MD2_Apamin: 33.075, TLR3_Apisimin:14.653, TLR4-MD2_Apisimin: 4.111, TLR3_Hymenoptaecin: 11.803, TLR4-MD2_Hymen optaecin: 3.749, TLR3_Melittin: 27.788, TLR4-MD2_Melittin: 22.697) (Figure 5 and Figure 6 and Table 6) for the different TLRs_BAMPs docked complexes. Furthermore, the covariance matrix score was used to confirm the compactness and stability of the TLR3/TLR4-MD2 docked complexes. These results were as follows: TLR3_Abaecin, 4.321; TLR3_Apamin, 6.342; TLR3_Apisimin, 3.271; TLR3_Hymenoptaecin, 6.231; TLR3_Melittin, 5.351; and TLR4-MD2_Abaecin, 5.312; TLR4-MD2_Apamin, 3.271; TLR4-MD2_Apisimin, 6.661; TLR4-MD2_Hymenoptaecin, 4.712; TLR4-MD2_Melittin, 6.331 (Figure 5 and Figure 6 and Table 6).

### 3.10. Secondary Structure Analysis of BAMPs-TLRs Complexes

The secondary structure evolution analysis was used to evaluate the stability of the TLRs_BAMPs docked complexes at an interval of 20 ns throughout the overall 100 ns MD simulation experiment (Figures S3 and S4). The TLR3/TLR4 receptor’s secondary structural components, including its α-helices and β-sheets, are observed as constantly preserved during the 100 ns MD simulation process, suggesting the receptors’ stability and consistency after binding to the BAMPs. Without undergoing any structural changes, the BAMPs were found to be firmly attached to the TLRs’ active sites, indicating the stability of the BAMPs complex with TLR3/TLR4-MD2 (Figures S3 and S4).

### 3.11. Immune Simulations Confirmed the Desired Immune Response from the BAMPs

**Figure 7:**
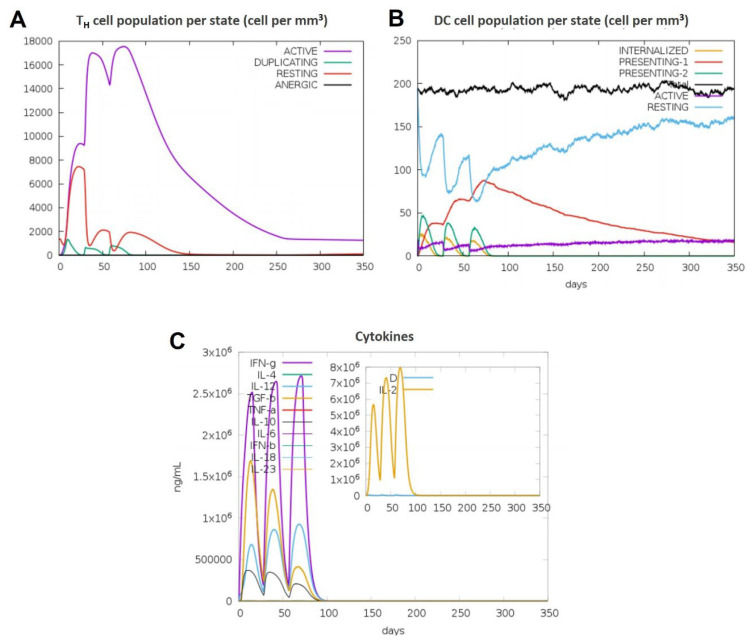
The in silico immune response of abaecin is analyzed using the C-Immsim server. (**A**) Total count per entity state of CD4 T helper cells. (**B**) Total count per entity state of DC cells. (**C**) Cytokine concentrations and interleukin in various states. All units are expressed in cells/mm^3^, in three successive immunological reactions.

**Figure 8:**
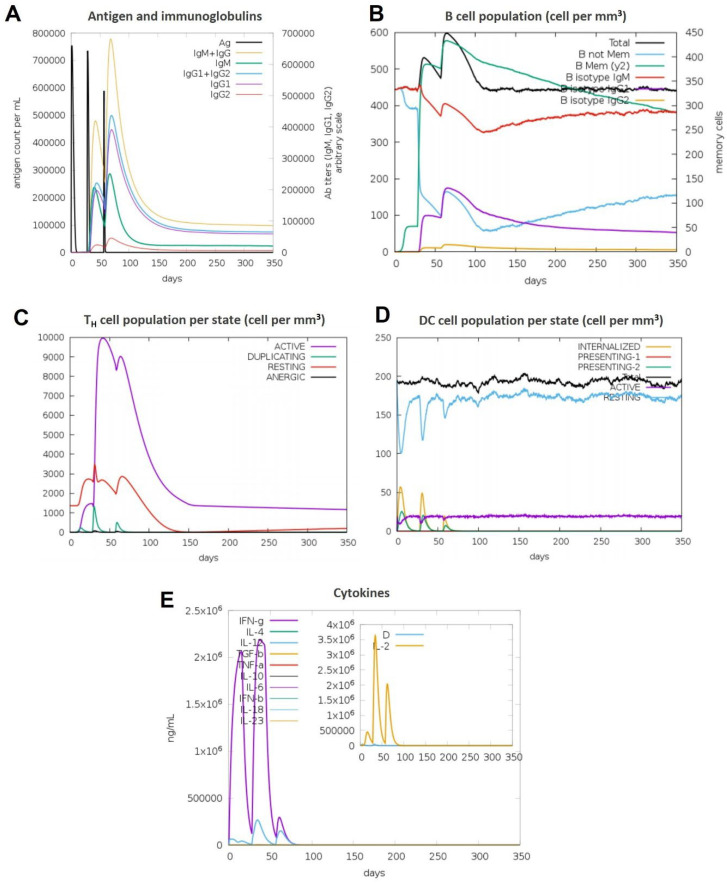
The in silico immune response of apamin is analyzed using the C-Immsim server. (**A**) Response of antibodies and antibody complexes to antigen. (**B**) Total count per entity state of B cells. (**C**) Total count per entity state of CD4 T helper cells. (**D**) Total count per entity state of DC cells. (**E**) Cytokine concentrations and interleukin in various states. All units are expressed in cells/mm^3^, in three successive immunological reactions.

**Figure 9:**
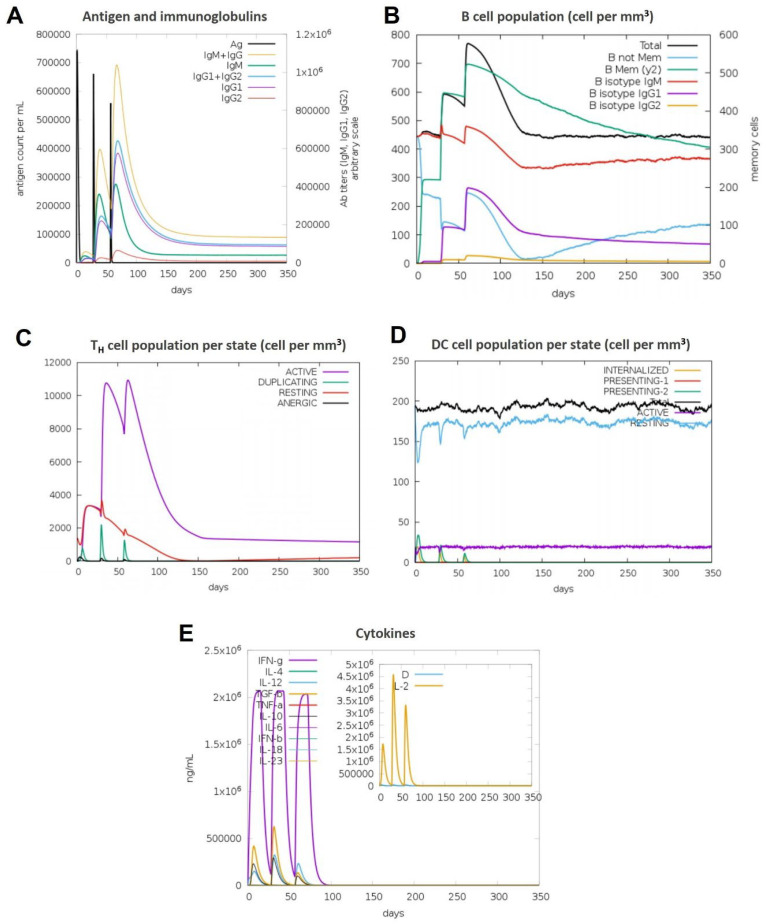
The in silico immune response of apisimin is analyzed using the C-Immsim server. (**A**) Response of antibodies and antibody complexes to antigen. (**B**) Total count per entity state of B cells. (**C**) Total count per entity state of CD4 T helper cells. (**D**) Total count per entity state of DC cells. (**E**) Cytokine concentrations and interleukin in various states. All units are expressed in cells/mm^3^, in three successive immunological reactions.

**Figure 10:**
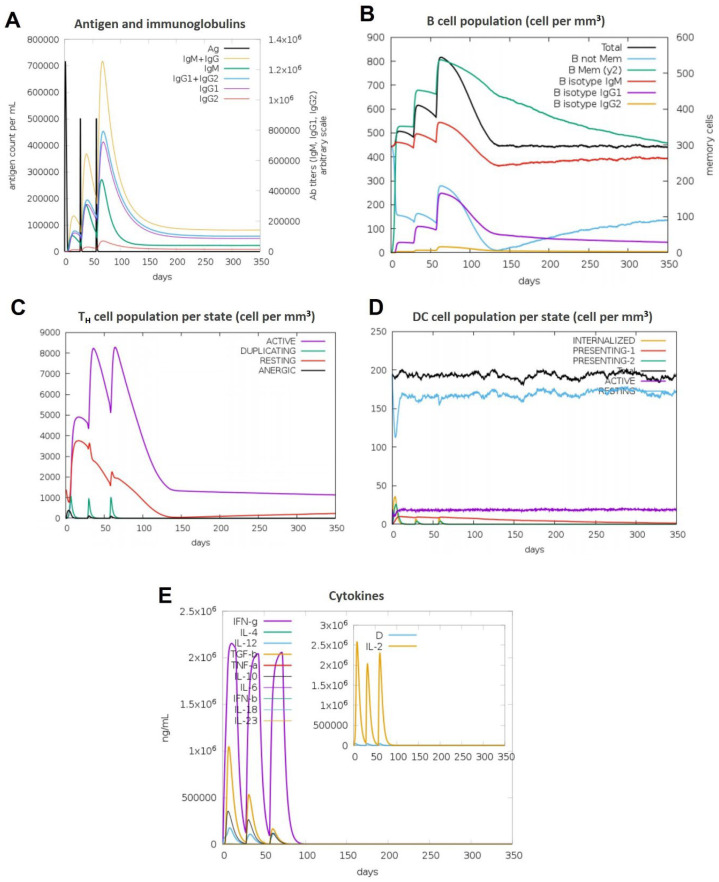
The in silico immune response of hymenoptaecin is analyzed using the C-Immsim server. (**A**) Response of antibodies and antibody complexes to antigen. (**B**) Total count per entity state of B cells. (**C**) Total count per entity state of CD4 T helper cells. (**D**) Total count per entity state of DC cells. (**E**) Cytokine concentrations and interleukin in various states. All units are expressed in cells/mm^3^, in three successive immunological reactions.

**Figure 11:**
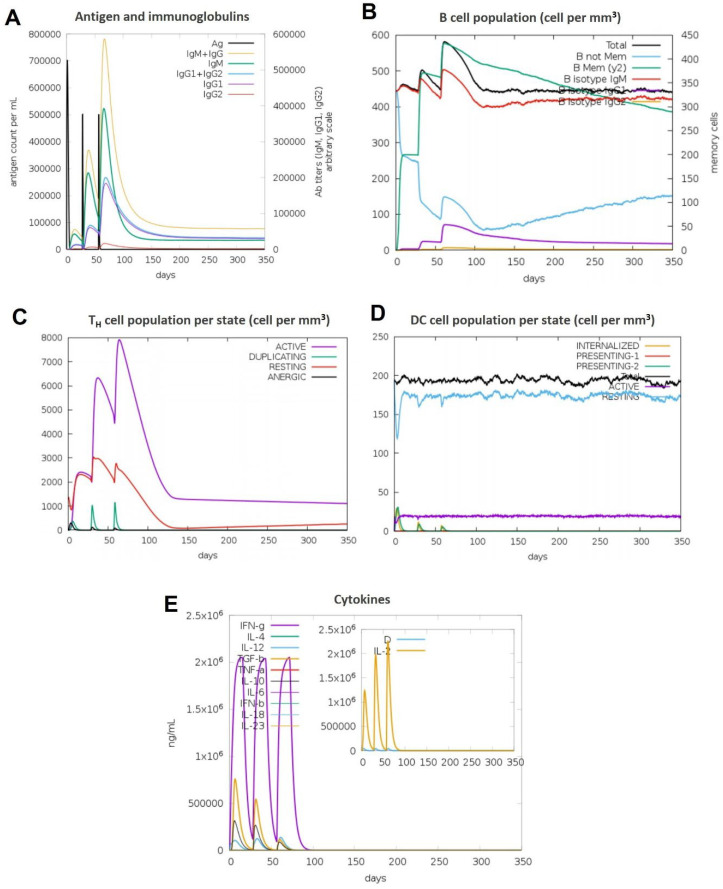
The in silico immune response of Mellitin is analyzed using the C-Immsim server. (**A**) Response of antibodies and antibody complexes to antigen. (**B**) Total count per entity state of B cells. (**C**) Total count per entity state of CD4 T helper cells. (**D**) Total count per entity state of DC cells. (**E**) Cytokine concentrations and interleukin in various states. All units are expressed in cells/mm^3^, in three successive immunological reactions.

Overall, after examining each immunogenic property separately, we used the C-IMMSIM server to run immune simulations to examine the total immune response of each chosen BAMPs. The C-IMMSIM server’s immune simulation results were in line with actual increased secondary and tertiary immune responses. The findings showed that both humoral and cellular immunological responses could be elicited by all of the chosen BAMPs (abaecin, apamin, apisimin, hymenoptaecin, and melittin) (Figure 7, Figure 8, Figure 9, Figure 10 and Figure 11). The data showed that for all of the selected BAMPs, the secondary and tertiary immune responses were higher than the primary reaction (Figure 7C, Figure 9E, Figure 10E and Figure 11E); however, the tertiary immune response for apamin (Figure 8E) is significantly lower. Following the three dosages of BAMPs, antigenic molecules were subsequently removed (Figure 7A, Figure 8A and Figure 9A); however, in the case of abaecin, this response is not shown. Curiously, the populations of B cells and T memory cells of some BAMPs similarly exhibit notable augmentation (Figure 8B,C, Figure 9B,C, Figure 10B,C and Figure 11C); the exception is abaecin, where a population increase was noted for T memory cells (Figure 7A) but not for B cells. Additionally, all five of the selected BAMPs (Figure 7C, Figure 8E, Figure 9E, Figure 10E and Figure 11E) show elevated amounts of cytokines such as IFN-γ and IL-2, which were critical in preventing viral multiplication and T cell-mediated immunity. Next, all of the selected BAMPs (Figure 7A, Figure 9A, Figure 10A and Figure 11A) showed a substantial increase in IgM and IgG antibody titers, except for abaecin. Additionally, several long-lasting B cell isotypes that did not respond to abaecin were seen, suggesting a significant shift in memory cells and B cell isotypes (Figure 7B, Figure 9B, Figure 10B and Figure 11B). Dendritic cells were also seen to be continuously proliferating (Figure 7B, Figure 9D, Figure 10D and Figure 11D). Overall findings indicated that all of the selected BAMPs might be useful as adjuvants, carrier proteins, candidates, or for other therapeutic uses in humans.

## 4. Discussion

HTL and CTL epitopes play a crucial role in the sequential development of an adaptive immune response against many microbial infections. It is well established that CTL and HTL epitopes are fundamentally involved in and significantly contribute to the immune response being escalated against different illnesses. During external and intracellular infections, respectively, MHC class I and class II recognition among the cytotoxic T cells and helper T cells is essential [52,53]. The CTL epitope prediction in a subset of BAMPs was performed in this study using the NETCTL 1.2 server, which identified three, five, three, and seven CTL epitopes in abacein, apisimin, hymenoptaecin, and melittin, respectively. Additionally, after initiating a sequence of chain reactions, helper T cells stimulate macrophages, cytotoxic T lymphocytes, and B cells, which promote an immunological response [54]. The HTL epitopes in the BAMPs are predicted and analyzed using the IEDB server. It is interesting to note that the HTL epitopes for all five BAMPs are hymenoptacein (3), apisimin (2), melittin (2), apamin (1), and abaecin (1). Low percentile scores of projected HTL epitopes that can effectively elicit a helper T cell response indicate that BAMP has high antigenic characteristics. The main component of humoral immune responses is B lymphocytes, which can be concurrently activated by helper T cells to initiate an effective immune response and stimulated for differentiation [53,55]. The BCPREDS service is utilized in this investigation to anticipate the B-cell epitopes in certain BAMPs. Except for abaecin, all four BAMPs (apamin, apisimin, hymenoptaecin, and melittin) had B-cell epitopes. The threshold scores of anticipated B-cell epitopes validate the immunogenic features of the BAMPs that were previously selected. Another significant immune system component that is crucial to the establishment of the immunological response is IFN-γ. Particularly in intracellular pathogenic infections, IFN-γ functions as a cytokine for natural killer cells and cytotoxic T lymphocytes, which then go on to trigger a particular immune response [31]. We predicted IFN-γ-inducing epitopes in specific BAMPs using the IFN epitope server. The only compounds reported to have two and one IFN-γ-inducing epitope, respectively, are hymenoptaecin and melittin. Hymenoptaecin’s two predicted epitopes have scores above the threshold value of 0.4, indicating that they have a strong ability to induce IFN-γ and trigger an immunological response. Based on a dataset of MHC class II binders that can activate T helper cells and induce IFN-γ, this prediction was made. Additionally, the ANTIGENpro server was used to predict the antigenicity of the selected BAMPs, and it was found to be an antigenic peptide with an antigenicity score of 0.362 to 0.7086 [34]. BAMPs were subjected to analysis using the VaxiJen server to verify their antigenicity. The results indicated that the antigenicity score of the BAMPs ranged between 0.3736 and 0.5031, both of which were higher than the default threshold value. BAMPs were found to be highly antigenic peptides by both antigenicity and IFN-γ prediction, indicating that they may elicit a stronger and longer-lasting immune response (0.9) [35]. Furthermore, BAMPs have an AllerTOP value of ≥0.5, indicating that they are non-allergenic. All of the selected BAMPs had multiple distinct epitopes, confirming their high immunogenicity and making them a prime choice for an adjuvant or carrier protein [56]. For the upcoming molecular docking and simulations investigations, the 3D structures of a subset of BAMPs that were obtained from databases or homology-modeled are examined and confirmed. Each chosen BAMP is docked against human TLR3 and TLR4-MD2 to assess the effectiveness of the binding. The establishment of an effective immune response is known to depend on TLR3 and TLR4-MD2 receptors [57,58]. Furthermore, TLRs trigger the production of inflammatory cytokines during infection and have a role in the activation of immune response in both innate and adaptive immunity [59,60]. Strong affinities between BAMPs and human TLRs were observed during molecular docking tests, indicating the possibility of a robust immunological response. We employed molecular dynamics and simulation experiments with docked complexes of TLRs/BAMPs to verify the stability, flexibility, and compactness by examining RMSD, RMSF, and Rg values, based on the docking results. Overall, the results of the simulations supported the compact complexes, reduced fluctuations, and structural stability of the BAMPs with TLRs [42]. Next, we used the C-ImmSim server to conduct in silico immune simulations to explore the overall immunological response of specific BAMPs in humans. These simulations demonstrate that BAMPs elicit a robust and sustained immune response [49,51]. Immune simulation data showed that during immune simulations of BAMPs, T cells, B cells, and primary antibodies (IgG, IgM) are generated.

## 5. Conclusions and Limitations

This study reveals that BAMPs possess favorable immunological features, including high antigenicity, diverse epitope content, and strong predicted binding to TLR3 and TLR4–MD2 receptors, supporting their potential role as adjuvants or carrier molecules in subunit vaccine design. Molecular docking, dynamics simulations, and MM-PBSA analyses consistently indicate stable receptor-peptide interactions and energetically favorable complexes, suggesting their capacity to elicit balanced humoral and cellular immune responses. Nevertheless, experimental validation, including peptide synthesis, binding and stability assays, cytotoxicity profiling, and in vivo immunogenicity studies, is required to substantiate the translational potential of these computational findings.

## Figures and Tables

**Table 1:** Cytotoxic T Lymphocyte (CTL) epitope prediction of the BAMPs from different honey bee species by using the NetCTL 1.2 Server.

BAMPs	Super Type	Epitopes		Cleavage	TAB	Combined Score
Abaecin: P15450	A1	ATICAAFAY	3	0.63	3.16	1.96
	B7	VPQPGRRPF		0.94	2.40	1.85
	B62	LLATICAAF		0.90	2.72	1.35
Apisimin: A0A2A3EK62	A1	LSPMTNNLY	5	0.88	3.10	2.92
	A3	ILRGESLNK		0.88	0.69	1.51
	A24	LYYVNTEQF		0.96	3.04	1.78
	B39	HYEGVQNIL		0.96	0.99	2.01
	B62	MQKMVNNDF		0.65	2.75	1.42
Hymenoptaecin: C7AHW3	A1	DIDYKHRVY	3	0.93	2.60	1.60
	A2	VLFCAIAYV		0.97	0.55	1.41
	B8	EPSRQHAGF		0.95	1.99	1.32
Melittin: I3RJI9A	A1	MVVYISYIY	7	0.97	3.08	1.35
	A2	FMVVYISYI		0.96	0.68	1.51
	A3	ALISWIKRK		0.92	0.55	1.32
	B8	VLKVLTTGL		0.61	1.18	1.52
	B44	PEAGIGAVL		0.94	0.21	1.54
	B58	TGLPALISW		0.87	0.62	1.60
	B62	FLVNVALVF		0.75	2.63	1.33

Combined score ≥ 1.25 threshold value for CTL epitopes.

**Table 2:** Helper T Lymphocytes (HTL) epitope prediction of BAMPs by the IEDB Server.

BAMPs	Epitopes	Percentile Rank	Number of HTL Epitopes
Abaecin: P15450	AAFAYVPLPNVPQPG	1.45	1
Apamin: A0A2A3EK62	ILITSYFVTPVMPCN	1.25	1
Apisimin: Q86BU7	LVSSIVSGANVSAVL	2.10	2
	VSDVSAKTSISAKAE	1.03	
Hymenoptaecin: C7AHW3	LFCAIAYVSAQAELE	0.90	2
	MDYVPSRFRRQDNPH	0.35	
Melittin: I3RJI9A	VYISFIYAAPEPEPA	0.70	2
	LPALISWIKRKRQQG	0.22	

Compounds with the least percentile rank show high affinity.

**Table 3:** B-Cell Epitope prediction of bee antimicrobial peptides by BCPREDS Server.

BAMPs	Epitopes	Score	No. of Epitopes
Apamin: A0A2A3EK62	YFVTPVMPCNCKAPETALCA	0.97	1
Apisimin: Q86BU7	LDCNTNSDTMVYIADEKGEG	0.94	10
	SPMTNNLYYSPVASTSLYYV	0.94	
	LVCLGIVCQGTTGNILRGES	0.93	
	KQVEIPHDVAVNATTGKGRL	0.93	
	LHEWKFFDYDFGSDERRQDA	0.92	
	TSNTFDYDPKFTKMTIDGES	0.87	
	ILNTRCENPDNDRTPFKISI	0.83	
	VSAKTSISVKGESNVDVVSQ	0.91	
	ISAKAESNVDVVSQINSLVS	0.88	
	KKVGDGGPLLQPYPDWSFAK	0.99	
Hymenoptaecin: C7AHW3	SEVQRGPGGRLPPYVGINGG	1	16
	NGMTGDAYGGVNIRPGQPAR	1	
	RRQERGSIVIQGTKEGRNRP	0.97	
	SEVQRGPGGRLSPYVGMNGG	1	
	TGDAYGGVNIRPGQPTRQHA	0.99	
	IRGQSEVQRGPGGRLSPYVG	1	
	NGMTGDAYGGVNIRSGQPAR	0.99	
	QAELEPEDYIPSRFRRQERG	0.96	
	KGTKEGRNRPSLDIDYKQRV	0.84	
	GDAYGGVSIRPGQPTRQHAG	0.99	
	IRGRSEVQRGPGGRLSPYVG	1	
	TGDAYGGVNIRPGQPTRQHA	0.99	
	GDAYGGVNIRPGQPTRQRAG	1	
	QAELEPEDYIPSRFRRQERG	0.96	
	KGTKEGRNRPSLDIDYKQRV	0.84	
	NGMTGDAYGGVNIRPGQPAR	1	
Melittin: I3RJI9A	APEPEPAPEAEAEADAEADP	1	3
	YAAPEPEPAPEPEAEADAEA	1	
	YAAPEPEPAPEAEAEADAEA	1	

Threshold scores ≥ 0.70 are considered as B-cell epitopes.

**Table 4:** IFN-γ epitope prediction of BAMPs using (https://webs.iiitd.edu.in/raghava/ifnepitope/predict.php, accessed on 6 October 2024).

BAMPs	Epitopes	Method	Score	Number of Epitopes
Hymenoptaecin: C7AHW3	VVVLSFELLHAPATV	SVM	0.48	2
	VVLSFELLHAPATVC	SVM	0.47	
Mellitin: I3RJI9A	MKVVIFIFALLATICAAFAYVPLPNV	SVM	0.35	1

≥0.4 threshold score for IFN-γ epitopes.

**Table 5:** Details of antigenicity and allergenicity profiles of bee antimicrobial peptides by using VaxiJen v2.0, ANTIGENpro from scratch protein predictor, and AllergenFP.

BAMPs	Antigenicity				Allergenicity	
	VaxiJen Score	Antigenic/Non-Antigenic	ANTIGENpro Score	Antigenic/Non-Antigenic	AllergenFP Tanimoto Similarity Index Score	Allergic/Non-Allergic
Abaecin: P15450	0.6327	Antigenic	0.484418	Antigenic	0.81	Non-allergen
Apamin: A0A2A3EK62	0.3800	Non-Antigenic	0.178642	Antigenic	0.77	Non-allergen
Apisimin: Q86BU7	0.4738	Antigenic	0.165312	Antigenic	0.85	Non-allergen
Hymenoptaecin: C7AHW3	0.8128	Antigenic	0.595802	Antigenic	0.78	Non-allergen
Melittin I3RJI9A	0.6664	Antigenic	0.353139	Antigenic	1.0	Allergen

Note: VaxiJen v2.0: >0.4, ANTIGENpro: ≤0.9, AllergenFP Tanimoto similarity index score ≤ 9.

**Table 6:** Details of mole-docking binding energy and RMSD, RMSF, Rg, number of H-bonds, Molecular mechanics Poisson-Boltzmann surface area (MMPBSA, ΔEMMPBSA) binding energy analysis for the TLR3 and TLR4 receptor complexes with *A. cerana* antimicrobial peptide calculated from molecular dynamics simulation trajectories.

Receptor	PatchDock		FireDock			H-Bond Analysis	
	ACE	Transformation	Global Energy (kcal/mol)	Attractive Vdw	Repulsive Vdw	No. of H-Bonds	Residues Involved in H-Bond Formation
**Molecular docking**							
Abaecin: P15450							
TLR3	−248.81	−2.33, −0.28, −0.02, 41.47, −26.3, 95.76	−30.11	−27.3	13.7	3	ILE661-PHE37, ASN659-PHE43, TRP660-PHE34
TLR4	−179.52	−1.82, −1.02, −2.93, −11.2, 47.7, 113.3	−22.51	−13.2	12.9	3	ARG31-HIS431, SER368-PRO45, LYS122-PHE37
Apamin: A0A2A3EK62							
TLR3	−631.67	1.03, −0.02, −0.73, −74.4, −47.4, −121.7	−35.2	−15.6	13.7	9	ASN517-ASN29, ASN520-GLN44, ARG544-GLN44, ARG544-GLN43, THR23-ASN517, CYS30-ASN517, LYS31-LYS467, LYS493-LYS31, PRO24-ASN541
TLR4	−315.77	−0.58, −0.14, 2.20, 123.3, 90.0, 19.7	−34.1	−23.9	13.43	7	HIS431-GLU34, LYS122-A:SER19, SER19-LYS122, CYS28-VAL93, LYS341-TYR20, PRO88-GLN44, GLY46-LYS125
Apisimin: Q86BU7							
TLR3	−165.50	1.03, 0.52, −2.20, 24.6, −25.7, 81.7	−30.7	−19.3	19.2	4	GLN208-ASN75, TYR283-ALA57, VAL39-HIS156, ALA61-TYR283
TLR4	−171.68	0.59, −0.56, 0.22, 74.7, 28.6, 39.6	−16.5	−10.6	11.8	3	ASN54-GLN21, SER360-ALA75, SER472-VAL39
Hymenoptaecin: C7AHW3							
TLR3	−149.82	0.32, −0.35, 0.89, 41.1, −17.4, −3.33	−55.3	−43.7	15.8	12	ARG489-ASP69, ASN517-GLY72, SER571-ASN76, SER571-ALA77, GLY573-TYR63, TYR63-LEU595, THR74-ASN515, GLY75-ASN517, ASN596-TYR63, PRO646-GLY80, GLY80-PHE644, ALA519-LEU59
TLR4	−92.91	1.84, 0.53, 1.79, 22.8, −30.7, 5.29	−50.31	−40.4	0.53	10	SER360-ASP60, ARG382-GLN51, ARG382-GLN51, ARG382-VAL52, ARG382-TYR68, TYR403-THR50, LYS477-THR46, GLN51-ASP428, TYR63-GLU143, ARG96-ASP60
Melittin: I3RJI9A							
TLR3	−111.68	−2.04, 0.80, 1.90, 205.6, −271.8, 75.9	−31.4	−33.9	21.5	7	LEU4:N-GLU460, ARG331-GLU34, LYS589-LEU56, SER282-ALA21, ASN328-SER17, HIS565-LEU52, THR53-GLU533
TLR4	−174.93	2.48, 0.27, −0.58, 28.8, 299.8, −16.1	−23.8	−13.1	20.8	3	SER472-GLU26, SER472-GLU26, HIS431-LEU4
Protein stability, flexibility, and compactness analysis by MD Simulation							
**Receptor**	**Avg. RMSD (nm)**	**Avg. RMSF (nm)**	**Avg. Rg (nm)**	**Eigenvalue of PC1**	**Eigenvalue of PC2**	**% Variance of PC1**	**% Variance of PC2**
Abaecin							
TLR3	0.29	0.15	1.70	15.6	3.87	80.1	19.8
TLR4	0.12	0.19	2.55	6.06	0.97	86.1	13.8
Apamin							
TLR3	0.30	0.17	3.18	28.8	3.98	87.8	12.1
TLR4	0.24	0.26	2.96	2.92	1.44	66.9	33.0
Apisimin							
TLR3	0.19	0.15	1.68	20.3	3.49	85.3	14.6
TLR4	0.17	0.21	3.93	34.0	1.46	95.8	4.11
Hymenoptaecin							
TLR3	0.40	0.09	2.48	21.0	2.82	88.1	11.8
TLR4	0.38	0.16	2.16	24.5	0.95	96.3	3.74
Melittin							
TLR3	0.19	0.12	2.39	16.7	6.43	72.2	27.7
TLR4	0.27	0.18	2.19	6.09	1.78	77.3	22.6
Molecular mechanics Poisson-Boltzmann surface area (MMPBSA, ΔEMMPBSA) binding energy analysis for the TLR3 and TLR4 receptor complexes with *A. cerana* AMPs calculated from MD simulation trajectories.							
**Receptor**	**van der Waals energy** **(E_vdW_, kJ/mol)**	**Electrostatic energy** **(E_elec_, kJ/mol)**	**Polar solvation energy** **(E_polar_, kJ/mol)**	**Solvent accessible surface area (SASA energy, E_nonpolar_, kJ/mol)**		**Total Binding energy (ΔE_MMPBSA_, kJ/mol)**	
Abaecin							
TLR3	−217.1 ± 11.1	−231.4 ± 32.4	−176.3 ± 14.3	−21.4 ± 1.01		−71.9	
TLR4	−171.8 ± 21.3	−134.3 ± 53.1	−233.5 ± 13.4	−22.9 ± 1.33		−67.2	
Apamin							
TLR3	−221.4 ± 23.6	−235.2 ± 12.3	−176.5 ± 23.4	−42.5 ± 1.56		−158.8	
TLR4	−162.3 ± 16.4	−241.1 ± 16.5	−221.5 ± 13.4	−32.1 ± 1.31		−70.8	
Apisimin							
TLR3	−217.4 ± 12.3	−314.1 ± 23.6	−261.6 ± 22.4	−23.6 ± 1.76		−63.0	
TLR4	−134.5 ± 10.2	−161.1 ± 17.5	−301.3 ± 21.2	−22.4 ± 1.85		−77.9	
Hymenoptaecin							
TLR3	−188.4 ± 21.1	−333.1 ± 53.4	−221.5 ± 11.3	−26.1 ± 1.54		−109.4	
TLR4	−164.3 ± 24.3	−211.4 ± 27.7	−331.5 ± 22.4	−31.5 ± 1.89		−109.1	
Melittin							
TLR3	−226.3 ± 17.4	−271.6 ± 26.4	−218.5 ± 12.3	−18.4 ± 1.43		−116.6	
TLR4	−321.4 ± 15.5	−26.4 ± 26.5	−231.6 ± 41.3	−11.6 ± 1.67		−117.1	

## Data Availability

The original contributions presented in this study are included in the article/Supplementary Material. Further inquiries can be directed to the corresponding author.

## Supplementary Materials

The following supporting information can be downloaded at: https://www.mdpi.com/article/10.3390/cimb48010081/s1.
