# Supplementary material for: Honey Bee AMPs as a Novel Carrier Protein for the Development of a Subunit Vaccine: An Immunoinformatic Approach
Source: Curr Issues Mol Biol. 2026 Jan 14;48(1):81. doi: 10.3390/cimb48010081 (PMC12840399; doi:10.3390/cimb48010081)

Honey Bee AMPs as a Novel Carrier Protein for the Development of a Subunit Vaccine: An Immunoinformatic Approach

Roy Dinata [1,2,3,*], Piyush Baindara [2,3], Chettri Arati [1]
and Guruswami Gurusubramanian [1,*]

[1] Department of Zoology, Mizoram University, Aizawl 796004, Mizoram, India
[2] Animal Science Research Center, Division of Animal Sciences, University of Missouri, Columbia, MO 65211, USA
[3] National Swine Testing Center, University of Missouri, Columbia, MO 65211, USA
* Correspondence: dinataroy9@gmail.com (R.D.); gurus64@yahoo.com (G.G.)

Table S1. Details of BAMPs Uniprot accession ID, amino acid sequences, and their host species.

Sl. No.	Uniprot ID	Peptide sequence	Species name
**Abaecin**			
**1**	P15450	MKVVIFIFALLATICAAFAYVPLPNVPQPGRRPFPTFPGQGPFNPKIKWPQGY	*Apis mellifera*
**2**	A0A2A3E7A0	MKVVIFIFALLATICAAFAYVPLPNIPQPGRRPFPTFPGQGPFNPKIKWPTMFYS	*Apis cerana cerana*
**3**	B9UK34	MKVVIFIFALLATVCAAFAYVPLPNIPPGRRPFPTFPGQGPFNPKIKWPQGY	*Apis cerana cerana*
**4**	B9UK38	MKVVIFIFALLATVCAAFAYVPLPNIPQPGRRPFPTFPGQGPFNPKIKWPQGY	*Apis cerana cerana*
**5**	B9UK35	MKVVIFIFALLATICAAFAYVPLPNIPQPGRRPFPTFPGQGPFNPKIRWPQGY	*Apis cerana cerana*
**6**	B9UK30	MKVVIFIFALLATICAAFAYVPLPNIPQPGRRPFPTFPGQGPFNPKIKWPQGY	*Apis cerana cerana*
**7**	B9UK29	MKVVIFIFALLATICAAFAYVPLPNIPPGRRPFPTFPGQGPFNPKIKWPQGY	*Apis cerana cerana*
**8**	D2YZ38	MKVVIFIFALLATICAAFAYVPLPNIPQPGRRPFPTFPGQGPFNPKIKWPQGY	*Apis cerana japonica*
**9**	Q8WSY9	CAAFAYVPLPNVPQPGRRPFPTFPGQGPFNPKI	*Apis mellifera*
**10**	A0A0N0BGD9	MKVVTFIFVLLATICAIFAIYVPSSNIPPQRRRPFPTFPGQGPFNPKINLPRVGIF	*Melipona quadrifasciata*
**Apamin**			
**11**	P01500	MISMLRCIYLFLSVILITSYFVTPVMPCNCKAPETALCARRCQQHG	*Apis mellifera*
**12**	Q86QT2	MISMLRCISLFLSVILITGYFVTPVMSCNCKAPETALCARRCQQHG	*Apis cerana cerana*
**13**	B7UUK0	MISMLRCIYLFLSVILITSYFVTPVMPCNCKAPETALCARRCQQHG	*Apis mellifera*
**14**	A0A2A3EK62	MISMLRCISLFLSVILITGYFVTPVMSCNCKAPETALCARRCQQHG	*Apis cerana cerana*
**15**	Q06602	KNFALAILVVTFVVAVFGNTNLDPPTRPTRLRREAKPEAEPGNNRPVYIPQPRPPHPRLRRE AEPEAEPGNNRPVYIPQPRPPHPRLRREAELEAEPGNNRPVYISQPRPPHPRLRREAEPEAEP GNNRPVYIPQPRPPHPRLRREAELEAEPGNNRPVYISQPRPPHPRLRREAEPEAEPGNNRPV YIPQPRPPHPRLRREAEPEAEPGNNRPVYIPQPRPPHPRLRREAEPEAEPGNNRPVYIPQPRP PHPRLRREAKPEAKPGNNRPVYIPQPRPPHPRI	*Apis mellifera*
**Apidaecin**			
**16**	Q06601	MKNFALAILVVTFVVAVFGNTNLDPPTRPARLRREAKPEAEPGNNRPIYIPQPRPPHPRLRR EAEPKAEPGNNRPIYIPQPRPPHPRLRREAESEAEPGNNRPVYIPQPRPPHPRLRREPEAEPG NNRPVYIPQPRPPHPRLRREPEAEPGNNRPVYIPQPRPPHPRI	*Apis mellifera*
**17**	P35581	MKNFALAILVVTFVVAVFGNTNLDPPTRPTRLRREAEPEAEPGNNRPVYIPQPRPPHPRLRR EAEPEAEPGNNRPVYIPQPRPPHPRLRREAEPEAEPGNNRPVYIPQPRPPHPRLRREAEPEAE PGNNRPVYIPQPRPPHPRI	*Apis mellifera*
**18**	A0A088AIG0	MKNFALAILVVTFVVAVFGNTNLDPPTRPARLRREAKPEAEPGNNRPVYIPQPRPPHPRLR REAEPEAEPGNNRPIYIPQPRPPHPRLRREAEPEAEPGNNRPVYIPQPRPPHPRLRRKAEPEA EPGNNRPVYIPQPRPPHPRLRREAEPEAEPGNNRPVYIPQPRPPHPRLRRKAEPEAEPGNNR	*Apis mellifera*

		PVYIPQPRPPHPRLRRKAEPEAEPGNNRPVYIPQPRPPHPRLRREAKPEAKPGNNRPVYIPQP<br>RPPHPRLRREAEPEAEPGNNRPVYIPQPRPPHPRLRREAKPEAEPGNNRPVYIPQPRPPHPRL<br>RREAEPEAEPGNNRPVYIPQPRPPHPRI	
**19**	B9UKB8	MKNFALAILVITFIVAVFGNVNPDPPPQPARIRREAEPEAEPGNNRPVYIPQPRPPHPRLRRE<br>AKPEAEPGNNRPVYIPQPRPPHPGLRREPEPEAEPSNNRPVYIPQPRPPHPRLRREAEPEAEA<br>GNNRPVYIPQPRPPHPRLRREAEPEAEPGNNRPVYIPQPRPPHPRI	*Apis cerana cerana*
**20**	B9UKB6	MKNFALAILVITFIVAVFGNVNPDPPPQPARICREAEPEAEPGNNRPVYIPQPRPPHPRLRRE<br>AKPEAEPGNNRPVYIPQPRPPHPGLRREPEPEAEPSNNRPVYISQPRPPHPRLRREAEPEAEA<br>GNNRPVYIPQPRPPHPRLRREAEPEAEPGINRPVYIPQPRPPHPRI	*Apis cerana cerana*
**21**	B9UKB5	MKNFALAILVITLIVAVFGNVNPDPPTQPARIRREAEPEAEPGNNRPVYIPQPRPPHPRLRRE<br>AKPEAEPGNNRPVYIPQPRPPHPRLRREPEPEAEPSNNRPVYILQPRPPHPRLRREAEPEAEA<br>GNNRPVYIPQPRPPHPRLRREAEPEAEPGNNRPVYIPQPRPPHPRI	*Apis cerana cerana*
**22**	B9UKB7	MKNFALAILVITFIVAVFGNANPDPPPQPARIRREAEPEAEPGNNRPVYIPQPRPPHPRLRRE<br>AKPEAEPGNNRPVYIPQPRPPHPRLRREPEPEAEPSNNRPVYIPQPRPPHPRLRREAEPEAEA<br>GNNRPVYIPQPRPPHPRLRREAEPEAEPGNNRPVYSPQPRPPHPRI	*Apis cerana cerana*
**23**	B9UKC1	MKNFALAILVITFIVAVFGNVNPDPPTQPARIRREAEPEAEPGNNRPVYIPQPRPPHPRLRRE<br>AKPEAEPGNNRPVYIPQPRPPHPRLRREPEPEAEPSNNRPVYIPQPRPPHPRLRREAEPEAEA<br>GNNRPVYIPQPRPPHPRLRREAEPEAEPGNNRPVYIPQPRPPHPRI	*Apis cerana cerana*
**24**	B9UKB4	MKNFALAILVITFIVAVFGNVNPDPPPQPARIRREAEPEAEPGNNRPVYIPQPRPPHPRLRRE<br>AKPEAEPGNNRPVYIPQPRPPHPRLRREPEPEAEPSNNRPVYIPQPRPPHPRLRREAEPEAEA<br>GNNRPVYIPQPRPPHPRLRREAEPEAEPGNNRPVYIPQPRPPHPRI	*Apis cerana cerana*
**25**	B9UKC0	MKNFALAILVITFIVAVFGNVNPDPPPQPARIRREAEPEAESGNNRPVYIPQPRPPHPRLRRE<br>AKPEAEPGNNRPVYIPQPRPPHPRLRREPEPEAEPSNNRPVYIPQPRPPHPRLRREAEPEAEA<br>GNNRPVYIPQPRPPHPRLRREAEPEAEPGNNRPIYIPQPRPPHPRI	*Apis cerana cerana*
**26**	A0A088A865	MKNFALAILVVTFVVAVFGNTNLDPPTRPTRLRREAKPEAEPGNNRPVYIPQPRPPHPRLR<br>REAEPEAEPGNNRPVYIPQPRPPHPRLRREAEPEAEPGNNRPVYIPQPRPPHPRLRREAEPEA<br>EPGNNRPVYIPQPRPPHPRLRREAKPEAEPGNNRPVYIPQPRPPHPRLRREAEPEAKPGNNR<br>PVYIPQPRPPHPRLRREAESEAEPGNNRPVYIPQPRPPHPRLRREPEAEPGNNRPVYIPQPRP<br>PHPRI	*Apis mellifera*
**27**	A0A2A3E8G9	MKNFALAILVITFIVAVFGNVNPDPPPQPARIRREAEPEAEPGNNRPVYIPQPRPPHPRLRRE<br>AKPEAEPGNNRPVYIPQPRPPHPRLRRKPEPEAEPGNNRPRLRREAEPEAEPGNNRPVYIPQ<br>PRPPHPRLRREAKPEAEPGNNRPVYIPQPRPPHPVNI	*Apis cerana cerana*
**28**	Q8WSY8	TRLRREAKPEAEPGNNRPVYIPQPRPPHPRLRREAEPEAEPGNNRPVYIPQPRPPHPRLRRE<br>AEPEAEPGNNRPVYIPQPRPPHPRLRREAEPEAEPGNNRPVYIPQPRPPHPRLRREAEPEAEP	*Apis mellifera*

		GNNRPVYIPQPRPPHPRLRREAKPEAEPGNNRPVYIPQPRPPHPRLRREAEPEAEPGNNRPV YIPQPRPPHPRI	
**29**	B9UKF6	MKLIVLVVFCAIAYVSAQAELEPEDYIPSRFRRQERGSIVIQGTKEGRNRPSLDIDYKQRVY DKNGMTGNAYGGVNIRPGQPTRQHAGFEFGKEYKNGFIRGQSEVQRGPGGRLSPYVGING GFRF	*Apis cerana cerana*
**30**	B9UKG0	MKLIVLVVFCAIAYVSAQAELEPEDYIPSRFRRQERGSIVIKGTKEGRNRPSLDIDYKQRVY DKNGMTGDAYGGVNIRPGQPARQHAGFEFGKEYKNGFIRGQSEVQRGPGGRLSPYVGIN GGFRF	*Apis cerana cerana*
**31**	B9UKG4	MKLIVLVVFCAIAYVSAQAELEPEDYIPSRFRRQERGSIVIQGTKEGRNRPSLDIDYKQRVY DKNGMTGDAYGGVNIRPGQPTRQHAGFEFGKEYKNGFIRGQSEVQRGPGGRLSPYVGING GFRF	*Apis cerana cerana*
**32**	B9UN30	MKLIVLVVFCAIAYVSAQAELEPEDYIPSRFRRQERGSIVIKGTKEGRNRPSLDIDYKQRVY DKNGMTGDAYGGVNIRPGQPTRQHAGFEFGKEYKNGFIRGQSEVQRGPGGRLSPYVGING GFRF	*Apis cerana cerana*
**33**	B9UN27	MKLIVLVVFCAIAYVSAQAELEPEDYIPSRFRRQERGSIVIKGTKEGRNRPSLDIDYKQRVY DKNGMTGDAYGGVNIRPGQPARQRAGFEFGKEYKNGFIRGQSEVQRGPGGRLSPYVGIN GGFRF	*Apis cerana cerana*
**34**	B9UN24	MKLIVLVVFCAIAYVSAQAELEPEDYIPSRFRRQERGSIVIQGTKEGRNRPSLDIDYKQRVY DKNGMTGDAYGGVNIRPGQPTRQHAGFEFGKEYKNGFIRGLSEVQRGPGGRLSPYVGING GFRF	*Apis cerana cerana*
**35**	B9UN22	MKLIVLVVFCAIAYVSAQAELEPEGYIPSRFRRQERGSIVIQGTKEGRNRPSLDIDYKQRVY DKNGMTGDAYGGVNIRPGQPARQHAGFEFGKEYKNGFIRGQSEVQRGPGGRLSPYVGIN GGFRF	*Apis cerana cerana*
**36**	B9UN18	MKLIVLVVFCAIAYASAQAELEPEDYIPSRFRQQERGSIVIQGTKEGRNRPSLDIDYKQRVY DKNGMTGDAYGGVNIRPGQPARQHAGFEFGKEYKDGFIRGQSEVQRGPGGRLSPYVGIN GGFRF	*Apis cerana cerana*
**37**	A0A0N0U6Q6	MKFIFAILVVTFAVATCLDTVTPSESLLISCDKTITKLSLQRRLRREADPEPEPKRPRIPHIPRP RPPQIPRPRPPHPRLRREADPEPEPDNRPDILIYIPPPGPPHTRLRREADPEPEPDNRPDILIYIP PPGPPHTRLRREADPEPEPDNRPDILIYIPPPGPPHTVSN	*Melipona quadrifasciata*
**Apisimin**			
**38**	Q8ISL8	MTRLFMLVCLGIVCQGTTGNILRGESLNKSLPILHEWKFFDYDFGSDERRQDAILSGEYDY KNNYPSDIDQWHDKIFVTMLRYNGVPSSLNVISKKVGDGGPLLQPYPDWSFAKYDDCSGI VSASKLAIDKCDRLWVLDSGLVNNTQPMCSPKLLTFDLTTSQLLKQVEIPHDVAVNATTG KGRLSSLAVQSLDCNTNSDTMVYIADEKGEGLIVYHNSDDSFHRLTSNTFDYDPKFTKMTI DGESYTAQDGISGMALSPMTNNLYYSPVASTSLYYVNTEQFRTSDYQQNDIHYEGVQNIL	*Apis mellifera*

		DTQSSAKVVSKSGVLFFGLVGDSALGCWNEHRTLERHNIRTVAQSDETLQMIASMKIKEALPHVPIFDRYINREYILVLSNKMQKMVNNDFNFDDVNFRIMNANVNELILNTRCENPDNDRTPFKISIHL	
**39**	O18330	MSKIVAVVVLAAFCVAMLVSDVSAKTSISVKGESNVDVVSQINSLVSSIVSGANVSAVLLAQTLVNILQILIDANVFA	*Apis mellifera*
**40**	A0A2A3EKN9	MSKIIAVVVLAAFCVAMLVSDVSAKTSISAKAESNVDVVSQINSLVSSIVAGANVSAVLLAQTLVNILQILIDANILIQR	*Apis cerana cerana*
**41**	Q86BU7	MSKIIAVVVLAAFCVAMLVSDVSAKTSISAKAESNVDVVSQINSLVSSIVSGANVSAVLLAQTLVNILQILIDANVFA	*Apis cerana cerana*
**Defensin**			
**42**	P17722	MKIYFIVGLLFMAMVAIMAAPVEDEFEPLEHFENEERADRHRRVTCDLLSFKGQVNDSACAANCLSLGKAGGHCEKGVCICRKTSFKDLWDKRFG	*Apis mellifera*
**43**	Q5J8R1	MKIYFIVGLLFMAMVAIMAAPVEDEFEPLEHFENEERADRHRRVTCDLLSFKGQVNDSACAANCLSLGKAGGHCEKGVCICRKTSFKDLWDKRFG	*Apis mellifera carnica*
**44**	Q5MQL3	MKFFVLFAILIAIVHASCASVPKVVYDGPIYELRQIEEENIEPDTELMDSNEPLLPLRHRRVTCDVLSWQSKWLSINHSACAIRCLAQRRKGGSCRNGVCICRK	*Apis mellifera*
**45**	A7L3U8	MKIYFIVAFLFMAMVAIMAAPVEDEFEPLEHPENEERTDRHRRVTCDLLSFKGQVNDSACAANCLSLGKAGGHCKNGVCICRKTSFKDLWDKRFG	*Apis cerana cerana*
**46**	B9UKD4	MKIYFIGAFLFMAMVAIMAAPVEDEFEPLEHPENEERTDRHRRVTCDLLSFKGQVNDSACAANCLSLGKAGGHCKNGVCICRKTSFKDLWDKRLG	*Apis cerana cerana*
**47**	B9UKD2	MKIYFIGAFLFMAMVAIMAAPVEDEFEPLEHPENEERTDRHRRVTCDLLSFKGQVNDSACAANCLSLGKAGGHCKNGVCICRKTSFKDFWDKRFG	*Apis cerana cerana*
**48**	B9UKE0	MKIYFIGAFLFMAMVAIMAASVEDEFEPLEHPENEERTDRHRRVTCDLLSFKGQVNDSACAANCLSLGKAGGHCKNGVCICRKTSFKDLWDKRFG	*Apis cerana cerana*
**49**	B9UKE3	MKIYFIGLLFMAMVAIMAAPVEDEFEPLEHPENEERTDRHRRVTCDLLSFKGQVNDSACAANCLSLGKAGGHCKNGVCICRKTSFKDLWDKRFG	*Apis cerana cerana*
**50**	B9UKC7	MKIYFIGAFLFMAMVAIMAAPVEDEFEPLEHPENEERTDRHRRVTCDLLSFKGQVNDSACAANCFSLGKAGGHCKNGVCICRKTSFKDFWDKRFG	*Apis cerana cerana*
**51**	B9UKD0	MKIYFIGAFLFMAMVAIMAAPVEDEFEPLEHPENEERTDRHRRVTCDLLSFKGQVNDSACAANCFSLGKAGGHCKNGVCICRKTSFKDLWDKRFG	*Apis cerana cerana*
**52**	B9UKD5	MKIYFIGAFLFMAMVAIMAAPVEDEFEPLEHPENEERTDRHRRVTCDLLSFKGQVNDSACAANCLSLGKAGGHCKNGVCICRKTSFKDLWDKRFG	*Apis cerana cerana*
**53**	B9UKF1	MKIYFIGAFLFMAMVAIMAAPVEDEFEPPEHPENEERTDRHRRVTCDLLSFKGQVNDSACAANCLSLGKAGGHCKNGVCICRKTSFKDLWDKRFG	*Apis cerana cerana*

**54**	B9UKC8	MKIYFIGAFLFMAMVAIMAAPVEDEFEPLEHPENEERTDRHRRVTCDLLSFEGQVNDSAC AANCLSLGKAGGHCKNGVCICRKTSFKDLWDKRFG	*Apis cerana cerana*
**55**	B9UKC9	MKIYFIGLLFMAMVAIMAAPVEDEFEPLEHPENEERTDRHRRVTCDLLSFEGQVNDSACA ANCLSLGKAGGHCKNGVCICRKTSFKDLWDKRFG	*Apis cerana cerana*
**56**	C7AHS9	MAAPVEDEFEPLEHPENEERTDRHRRVTCDLLSFKGQVNDSACAANCLSLGKAGGHCKN GVCICRKTSFKDLWDKRFG	*Apis cerana*
**57**	B9UKD3	MKIYFIGAFLFMAMVALMAAPFEDEFEPLERPENEERTDRHRRVTGDLLSFKGQVNDSAC AATCLSLGKAGGHCKNGVCICRKPSFKDLWDKRFG	*Apis cerana cerana*
**58**	A0A387IGF2	PLEHFETEERTDRHRRVTCDLLSFKGQINDSACAANCLSLGKAGGHCKNGVCICRKTSFKE LWDKR	*Apis dorsata*
**59**	A0A3B1EZ66	EPLEHPENEERTDRHRRVTCDLLSFKGQVNDSACAANCLSLGKAGGHCKNGVCICRKTSF KDLWDKR	*Apis cerana*
**60**	A0A2A3E8B7	MKFFVLFAILVAVVYASCASVSQDVYDEPIYDLRQIEEENIEPGTELMDSNEPLLLRHRRVT CDVLSWQSKWLSINHSACAIRCLAKRRKGGRCKNGVCICRK	*Apis cerana cerana*
**61**	D3KYH2	MKFFVLFAILVAIVYASCASVSQVVYDEPIYDLRQIEEENIEPDTELMDSNEPLLLRHRRVT CDVLSWQSKWLSINHSACAIRCLAKRRKGGRCKNGVCICRK	*Apis cerana japonica*
**62**	D3KYH1	MKFFVLFAILVAIVYASCASVSQVVYDEPIYDLRQIEEENIEPDTELMDSNEPLLLRHRRVT CDVLSWQSKWLSINHSSCAIRCLAKRRKGGRCKNGVCICRK	*Apis cerana japonica*
**63**	A0A0M9A1F6	LGEIEGTNDEATETVGDDSTDLAPLRHRRVTCDVLSFQSKWLSVNHSACAVRCLAQRRKG GSCRNGVCVCRK	*Melipona quadrifasciata*
**64**	A0A0N0U626	MVKIYFLVALLFVAVAATVAVPVVLATFELMITRLWTFKYLLDNLEEYEPFELHGVEERA DRQRRVTCDLLSGFVEHSACAANCLSMGKAGGRCENGICICRKTTFKELWDKRFG	*Melipona quadrifasciata*
**Hymenoptaecin**			
**65**	B9UKG1	MKFIVLVLFCAVAYVSAQAELEPEDTMDYIPTRFRRQERGSIVIQGTKEGKSRPSLDIDYKQ RVYDKNGMTGDAYGGLNIRPGQPSRQHAGFEFGKEYKNGFIKGQSEVQRGPGGRLSPYF GINGGFRF	*Apis mellifera*
**66**	B9UKH2	MKLIVLVVFCAIAYVSAQAELEPEDYIPSRFRRQERGSIVIQGTKEGRNRPSLDIDYKQRVY DKNGMTGDAYGGVNIRPGQPTRQHAGFEFGKEYKNGFIRGQSEVQRGPGGRLSPYVGMN GGFRF	*Apis cerana cerana*
**67**	B9UKH1	MKLIVLVVFCAIAYVSAQAELEPEDYIPSRFRRQERGSIVIKGTKEGRNRPSLDIDYKQRVY DKNGMTGDAYGGVNIRSGQPARQHAGFEFGKEYKNGFIRGQSEVQRGPGGRLSPYVGIN GGFRF	*Apis cerana cerana*
**68**	B9UKF8	MKLIVLVVFCAIAYVSAQAELEPEDYIPSRFRRQERGSIVIQGTKEGRNRPSLDIDYKQRVY DKNGMTGDAYGGVSIRPGQPTRQHAGFEFGKEYKNGFIRGQSEVQRGPGGRLSPYVGING GFRF	*Apis cerana cerana*

**69**	B9UKF9	MKLIVLVVFCAIAYVSAQAELEPEDYIPSRFRRQERGSIVIQGTKEGRNRPSLDIDHKQRVY DKNGMTGDAYGGVNIRPGQPTRQHAGFEFGKEYKNGFIRGRSEVQRGPGGRLSPYVGING GFRF	*Apis cerana cerana*
**70**	B9UKG3	MKLIVLVVFCAIAYVSAQAELEPEDYIPSRFRRQERGSIVIKGTKEGRNRPSLDIDYKQRVY DKNGMTGDAYGGVNIRPGQPTRQRAGFEFGKEYKNGFIRGQSEVQRGPGGRLSPYVGING GFRF	*Apis cerana cerana*
**71**	B9UKG2	MKLIVLVVFCAIAYVSAQAELEPEDYIPSRFRRQERGSIVIQGTKEGRNRPSLDIDYKQRVY DKNGMTGDAYGGVNIRPGQPARQHAGFEFGKEYKNGFIRGQSEVQRGPGGRLSPYVGIN GGFRF	*Apis cerana cerana*
**72**	A0A2A3ECM2	MKLIVLVVFCAIAYVSAQAELEPEDYIPSRFRRQERGSIVIKGTKEGRNRPSLDIDYKQRVY DKNGMTGDAYGGVNIRPGQPARQHAGFEFGKEYKNGFIRGQSEVQRGPGGRLSPYVGIN VMHLFRFDLINL	*Apis cerana cerana*
**73**	C7AHW4	MKFIVLVLFCAIAYVSAQEELELEDTMDYIPTRFRRQDNPHRGSIVIQGTKEGRNRPSLDVD YKHRVYDKNGMTGNAYGGVNIRPGQPSRQHAGFEFGKEYKNGFIKGQSEVQR	*Apis dorsata*
**74**	C7AHW3	MKFIVLVLFCAIAYVSAQAELEPEDTMDYVPSRFRRQDNPHRGSITVQGTQVGRNRPSLDI DYKHRVYDKNGMTGNAYGGVNIRPGEPSRQHAGFDFGKEYKNGFIRGQGEVQR	*Apis andreniformis*
**75**	V9IC30	MKLIVLVVFCAIAYVSAQAELEPEDYIPSRFRRQERGSIVIQGTKEGRNRPSLDIDYKQRVY DKNGMTGDAYGGVNIRPGQPARQHAGFEFGKEYKNGFIRGQSEVQRGPGGRLSPYVGIN GGFRF	*Apis cerana*
		**Melittin**	
**76**	P01501	MKFLVNVALVFMVVYISYIYAAPEPEPAPEPEAEADAEADPEAGIGAVLKVLTTGLPALIS WIKRKRQQG	*Apis mellifera*
**77**	Q8LW54	MKFLVNVALVFYGRVHFLHLCVHFLHLWAPEPEPAPEAEAEADAEADPEAGIGAVLKVL TTGLPALISWIKRKRQQG	*Apis cerana*
**78**	P0DPR9	MKFLVNVALVFMVVYISYIYAAPEPEPAPEPEAEADAEADPEAGIGAVLKVLTTGLPALIN WIKRKRQQG	*Apis cerana*
**79**	P01502	GIGAILKVLSTGLPALISWIKRKRQE	*Apis florea*
**80**	P01504	GIGAILKVLATGLPTLISWIKNKRKQ	*Apis dorsata*
**81**	P68407	MKFLVNVALVFMVVYISFIYAAPEPEPAPEAEAEADAEADPEAGIGAVLKVLTTGLPALIS WIKRKRQQG	*Apis cerana cerana*
**82**	I3RJI9	MKFLVNVALVFMVVYISYIYAAPEPEPAPEPEAEADAEADPEAGIGAVLKVLTTGLPALIS WIKRKRQQG	*Apis mellifera carnica*

Table S2. Tertiary structure prediction of bee Antimicrobial peptide by homology modeling.

Sequence ID	Favored regions	Additional allowed region	Generously allowed region	Disallowed Regions	Total number of residues
**1) Abaecin**					
AOA2A3E7A0	90.90	9.10	0.0	0.0	100
B9UK29	80.00	20.00	0.0	0.0	100
B9UK30	90.90	9.100	0.0	0.0	100
B9UK34	80.00	20.00	0.0	0.0	100
B9UK35	90.90	9.10	0.0	0.0	100
B9UK38	90.90	9.10	0.0	0.0	100
P15450	90.90	9.10	0.0	0.0	100
Q8WSY9	76.50	23.50	0.0	0.0	100
**2) Apamin**					
A0A2A3EK62	100.0	0.00	0.0	0.0	100
B7UUK0	95.70	4.30	0.0	0.0	100
P01500	95.70	4.30	0.0	0.0	100
Q86QT2	100.0	0.00	0.0	0.0	100
**3) Apidiaecin**					
A0A0N0U6Q6	78.60	14.30	7.1	0.0	100
A0A088A4M2	85.70	9.50	0.0	4.8	100
A0A088A865	81.80	18.20	0.0	0.0	100
A0A088AIG0	81.20	18.80	0.0	0.0	100
B9UKB5	69.70	30.30	0.0	0.0	100
B9UKC1	66.70	33.30	0.0	0.0	100
P35581	82.40	17.60	0.0	0.0	100
Q8WSY8	72.70	27.30	0.0	0.0	100
**4) Apisimin**					
A0A2A3EKN9	100.0	0.00	0.0	0.0	100
O18330	84.30	14.20	0.5	1.0	100
Q8ISL8	100.0	0.00	0.0	0.0	100
Q86BU7	100.0	0.00	0.0	0.0	100
**5) Defensin**					
A0A0M9A1F6	73.70	26.30	0.0	0.0	100

A0A0N0U626	83.90	16.10	0.0	0.0	100
A0A2A3E8B7	73.70	26.30	0.0	0.0	100
A0A3B1EZ66	78.80	21.20	0.0	0.0	100
A0A387IGF2	78.80	21.20	0.0	0.0	100
A7L3U8	78.80	18.20	0.0	3.0	100
B9UKC7	78.80	18.20	0.0	3.0	100
B9UKC8	78.80	18.20	0.0	3.0	100
B9UKC9	78.80	18.20	0.0	3.0	100
B9UKD0	78.80	18.20	0.0	3.0	100
B9UKD2	78.80	18.20	0.0	3.0	100
B9UKD3	81.20	18.80	0.0	0.0	100
B9UKD4	78.80	18.20	0.0	3.0	100
B9UKD5	78.80	18.20	0.0	3.0	100
B9UKE0	78.80	18.20	0.0	3.0	100
B9UKE3	78.80	18.20	0.0	3.0	100
B9UKF1	78.80	18.20	0.0	3.0	100
C7AHS9	78.80	21.20	0.0	0.0	100
D3KYH1	73.70	26.30	0.0	0.0	100
D3KYH2	73.70	26.30	0.0	0.0	100
P17722	78.80	21.20	0.0	0.0	100
Q5J8R1	78.80	21.20	0.0	0.0	100
Q5MQL3	73.70	26.30	0.0	0.0	100
**6) Hymenoptaecin**					
C7AHW3	96.70	3.30	0.0	0.0	100
**7) Melittin**					
I3RJI9	97.60	2.40	0.0	0.0	100
P0DPR9	97.60	2.40	0.0	0.0	100
P01501	97.60	2.40	0.0	0.0	100
P68407	100.0	0.00	0.0	0.0	100
Q8LW54	89.70	10.30	0.0	0.0	100

Table S3. Computation of interacting amino acids, distance, bond category, bond type, and donor: acceptor chemistry.

**Between TLR3 and the top hit BAMPs complex by Firedock**							
**Receptor–Ligand Complex**	**Distance**	**Bond Category**	**Bond Type**	**From**	**From Chemistry**	**To**	**To Chemistry**
**Abaecin (P15450)**							
A:ILE661:CA - A:PHE37:O	3.36457	Hydrogen Bond	Carbon Hydrogen Bond	A:ILE661:CA	H-Donor	A:PHE37:O	H-Acceptor
A:ASN659:ND2 - A:PHE43	4.09872	Hydrogen Bond	Pi-Donor Hydrogen Bond	A:ASN659:ND2	H-Donor	A:PHE43	Pi-Orbitals
A:TRP660 - A:PHE34	4.97222	Hydrophobic	Pi-Pi Stacked	A:TRP660	Pi-Orbitals	A:PHE34	Pi-Orbitals
A:CYS651 - A:ARG31	3.94754	Hydrophobic	Alkyl	A:CYS651	Alkyl	A:ARG31	Alkyl
A:PHE34 - A:MET642	5.48854	Hydrophobic	Pi-Alkyl	A:PHE34	Pi-Orbitals	A:MET642	Alkyl
A:PHE37 - A:VAL658	4.49074	Hydrophobic	Pi-Alkyl	A:PHE37	Pi-Orbitals	A:VAL658	Alkyl
**Apamin (A0A2A3EK62)**							
A:ASN517:ND2 - A:ASN29:O	2.5821	Hydrogen Bond	Conventional Hydrogen Bond	A:ASN517:ND2	H-Donor	A:ASN29:O	H-Acceptor
A:ASN520:ND2 - A:GLN44:OE1	2.3619	Hydrogen Bond	Conventional Hydrogen Bond	A:ASN520:ND2	H-Donor	A:GLN44:OE1	H-Acceptor
A:ARG544:NE - A:GLN44:O	2.38792	Hydrogen Bond	Conventional Hydrogen Bond	A:ARG544:NE	H-Donor	A:GLN44:O	H-Acceptor
A:ARG544:NH2 - A:GLN43:O	2.53997	Hydrogen Bond	Conventional Hydrogen Bond	A:ARG544:NH2	H-Donor	A:GLN43:O	H-Acceptor
A:THR23:OG1 - A:ASN517:OD1	2.53386	Hydrogen Bond	Conventional Hydrogen Bond	A:THR23:OG1	H-Donor	A:ASN517:OD1	H-Acceptor
A:CYS30:SG - A:ASN517:O	3.71807	Hydrogen Bond	Conventional Hydrogen Bond	A:CYS30:SG	H-Donor	A:ASN517:O	H-Acceptor
A:LYS31:NZ - A:LYS467:O	3.03822	Hydrogen Bond	Conventional Hydrogen Bond	A:LYS31:NZ	H-Donor	A:LYS467:O	H-Acceptor
A:LYS493:CE - A:LYS31:O	2.35674	Hydrogen Bond	Carbon Hydrogen Bond	A:LYS493:CE	H-Donor	A:LYS31:O	H-Acceptor
A:PRO24:CD - A:ASN541:OD1	3.28969	Hydrogen Bond	Carbon Hydrogen Bond	A:PRO24:CD	H-Donor	A:ASN541:OD1	H-Acceptor

A:MET26:SD - A:ASN515:OD1	2.61817	Other	Sulfur-X	A:MET26:SD	Sulfur	A:ASN515:OD1	O,N,S
A:MET26:SD - A:HIS539	4.02314	Other	Pi-Sulfur	A:MET26:SD	Sulfur	A:HIS539	Pi-Orbitals
A:ALA491 - A:LYS31	3.27366	Hydrophobic	Alkyl	A:ALA491	Alkyl	A:LYS31	Alkyl
A:LYS493 - A:PRO33	4.36836	Hydrophobic	Alkyl	A:LYS493	Alkyl	A:PRO33	Alkyl
A:LYS493 - A:LEU37	4.60618	Hydrophobic	Alkyl	A:LYS493	Alkyl	A:LEU37	Alkyl
A:ALA519 - A:ARG41	4.27682	Hydrophobic	Alkyl	A:ALA519	Alkyl	A:ARG41	Alkyl
A:VAL25 - A:LEU595	4.96777	Hydrophobic	Alkyl	A:VAL25	Alkyl	A:LEU595	Alkyl
A:HIS539 - A:VAL25	5.36076	Hydrophobic	Pi-Alkyl	A:HIS539	Pi-Orbitals	A:VAL25	Alkyl
A:HIS45 - A:ALA519	5.13099	Hydrophobic	Pi-Alkyl	A:HIS45	Pi-Orbitals	A:ALA519	Alkyl
**Apisimin (Q86BU7)**							
A:GLN208:NE2 - C:ASN75:O	3.39656	Hydrogen Bond	Conventional Hydrogen Bond	A:GLN208:NE2	H-Donor	C:ASN75:O	H-Acceptor
A:TYR283:OH - C:ALA57:O	3.21466	Hydrogen Bond	Conventional Hydrogen Bond	A:TYR283:OH	H-Donor	C:ALA57:O	H-Acceptor
C:VAL39:N - A:HIS156:NE2	3.37911	Hydrogen Bond	Conventional Hydrogen Bond	C:VAL39:N	H-Donor	A:HIS156:NE2	H-Acceptor
C:ALA61:N - A:TYR283:OH	2.81452	Hydrogen Bond	Conventional Hydrogen Bond	C:ALA61:N	H-Donor	A:TYR283:OH	H-Acceptor
A:MET278 - C:ILE49	5.36957	Hydrophobic	Alkyl	A:MET278	Alkyl	C:ILE49	Alkyl
C:VAL50 - A:MET278	4.25394	Hydrophobic	Alkyl	C:VAL50	Alkyl	A:MET278	Alkyl
C:ALA74 - A:LYS182	5.28585	Hydrophobic	Alkyl	C:ALA74	Alkyl	A:LYS182	Alkyl
A:HIS156 - C:VAL38	4.47271	Hydrophobic	Pi-Alkyl	A:HIS156	Pi-Orbitals	C:VAL38	Alkyl
A:HIS156 - C:VAL39	4.95436	Hydrophobic	Pi-Alkyl	A:HIS156	Pi-Orbitals	C:VAL39	Alkyl
A:TYR283 - C:ALA61	5.48795	Hydrophobic	Pi-Alkyl	A:TYR283	Pi-Orbitals	C:ALA61	Alkyl
A:PHE304 - C:VAL55	5.35628	Hydrophobic	Pi-Alkyl	A:PHE304	Pi-Orbitals	C:VAL55	Alkyl
A:TYR307 - C:ALA57	4.96611	Hydrophobic	Pi-Alkyl	A:TYR307	Pi-Orbitals	C:ALA57	Alkyl
A:TYR307 - C:LEU60	5.26033	Hydrophobic	Pi-Alkyl	A:TYR307	Pi-Orbitals	C:LEU60	Alkyl

**Hymenoptaecin (C7AHW3)**							
A:ARG489:NH1 - A:ASP69:OD1	4.81551	Electrostatic	Attractive Charge	A:ARG489:NH1	Positive	A:ASP69:OD1	Negative
A:ASN517:ND2 - A:GLY72:O	2.47568	Hydrogen Bond	Conventional Hydrogen Bond	A:ASN517:ND2	H-Donor	A:GLY72:O	H-Acceptor
A:SER571:OG - A:ASN76:OD1	3.36745	Hydrogen Bond	Conventional Hydrogen Bond	A:SER571:OG	H-Donor	A:ASN76:OD1	H-Acceptor
A:SER571:OG - A:ALA77:O	3.3671	Hydrogen Bond	Conventional Hydrogen Bond	A:SER571:OG	H-Donor	A:ALA77:O	H-Acceptor
A:GLY573:N - A:TYR63:OH	3.08658	Hydrogen Bond	Conventional Hydrogen Bond	A:GLY573:N	H-Donor	A:TYR63:OH	H-Acceptor
A:TYR63:OH - A:LEU595:O	2.16304	Hydrogen Bond	Conventional Hydrogen Bond	A:TYR63:OH	H-Donor	A:LEU595:O	H-Acceptor
A:THR74:OG1 - A:ASN515:O	2.42986	Hydrogen Bond	Conventional Hydrogen Bond	A:THR74:OG1	H-Donor	A:ASN515:O	H-Acceptor
A:GLY75:N - A:ASN517:OD1	2.28177	Hydrogen Bond	Conventional Hydrogen Bond	A:GLY75:N	H-Donor	A:ASN517:OD1	H-Acceptor
A:ASN596:CA - A:TYR63:OH	3.11921	Hydrogen Bond	Carbon Hydrogen Bond	A:ASN596:CA	H-Donor	A:TYR63:OH	H-Acceptor
A:PRO646:CD - A:GLY80:OXT	3.32882	Hydrogen Bond	Carbon Hydrogen Bond	A:PRO646:CD	H-Donor	A:GLY80:OXT	H-Acceptor
A:GLY80:CA - A:PHE644:O	3.65647	Hydrogen Bond	Carbon Hydrogen Bond	A:GLY80:CA	H-Donor	A:PHE644:O	H-Acceptor
A:ALA519 - A:LEU59	3.54923	Hydrophobic	Alkyl	A:ALA519	Alkyl	A:LEU59	Alkyl
**Melittin (I3RJI9A)**							
C:LEU4:N - A:GLU460:OE1	2.88064	Hydrogen Bond;Electrostatic	Salt Bridge	C:LEU4:N	H-Donor	A:GLU460:OE1	H-Acceptor
A:ARG331:NH2 - C:GLU34:OE1	4.36167	Electrostatic	Attractive Charge	A:ARG331:NH2	Positive	C:GLU34:OE1	Negative
A:LYS589:NZ - C:LEU56:O	2.50189	Electrostatic	Attractive Charge	A:LYS589:NZ	Positive	C:LEU56:O	Negative
A:SER282:OG - C:ALA21:O	3.16283	Hydrogen Bond	Conventional Hydrogen Bond	A:SER282:OG	H-Donor	C:ALA21:O	H-Acceptor

A:ASN328:ND2 - C:SER17:OG	3.39835	Hydrogen Bond	Conventional Hydrogen Bond	A:ASN328:ND2	H-Donor	C:SER17:OG	H-Acceptor
A:HIS565:ND1 - C:LEU52:O	3.0772	Hydrogen Bond	Conventional Hydrogen Bond	A:HIS565:ND1	H-Donor	C:LEU52:O	H-Acceptor
C:THR53:CA - A:GLU533:OE1	3.34948	Hydrogen Bond	Carbon Hydrogen Bond	C:THR53:CA	H-Donor	A:GLU533:OE1	H-Acceptor
A:GLU363:OE1 - C:PHE11	3.95494	Electrostatic	Pi-Anion	A:GLU363:OE1	Negative	C:PHE11	Pi-Orbitals
C:GLU42:OE2 - A:TYR462	4.75527	Electrostatic	Pi-Anion	C:GLU42:OE2	Negative	A:TYR462	Pi-Orbitals
C:LEU56:O - A:HIS565	4.71486	Electrostatic	Pi-Anion	C:LEU56:O	Negative	A:HIS565	Pi-Orbitals
A:LYS330 - C:VAL14	5.14369	Hydrophobic	Alkyl	A:LYS330	Alkyl	C:VAL14	Alkyl
A:MET486 - C:LEU4	5.19973	Hydrophobic	Alkyl	A:MET486	Alkyl	C:LEU4	Alkyl
A:ILE534 - C:LEU49	5.1194	Hydrophobic	Alkyl	A:ILE534	Alkyl	C:LEU49	Alkyl
A:ILE534 - C:LEU52	3.82111	Hydrophobic	Alkyl	A:ILE534	Alkyl	C:LEU52	Alkyl
A:TYR283 - C:ALA22	4.56341	Hydrophobic	Pi-Alkyl	A:TYR283	Pi-Orbitals	C:ALA22	Alkyl
A:HIS359 - C:VAL10	3.96998	Hydrophobic	Pi-Alkyl	A:HIS359	Pi-Orbitals	C:VAL10	Alkyl

Table S4. Computation of interacting amino acids, distance, bond category, bond type, and donor: acceptor chemistry.

**Between the TLR4 receptor and the top hit AMPs complex by Firedock**							
**Receptor–Ligand Complex**	**Distance**	**Bond Category**	**Bond Type**	**From**	**From Chemistry**	**To**	**To Chemistry**
**Abaecin (P15450)**							
A:ARG32:NH2 - C:GLU92:OE2	4.10029	Electrostatic	Attractive Charge	A:ARG32:NH2	Positive	C:GLU92:OE2	Negative
A:ARG31:NH1 - A:HIS431:O	2.35082	Hydrogen Bond	Conventional Hydrogen Bond	A:ARG31:NH1	H-Donor	A:HIS431:O	H-Acceptor
A:SER368:CB - A:PRO45:O	2.83479	Hydrogen Bond	Carbon Hydrogen Bond	A:SER368:CB	H-Donor	A:PRO45:O	H-Acceptor
A:ALA366:CB - A:PHE43	3.33797	Hydrophobic	Pi-Sigma	A:ALA366:CB	C-H	A:PHE43	Pi-Orbitals

A:GLY363:C,O;GLY364:N - A:PHE37	5.36846	Hydrophobic	Amide-Pi Stacked	A:GLY363:C,O;GLY 364:N	Amide	A:PHE37	Pi-Orbitals
C:LYS91 - A:PRO29	3.80454	Hydrophobic	Alkyl	C:LYS91	Alkyl	A:PRO29	Alkyl
**Apamin (A0A2A3EK62)**							
A:ARG41:NH2 - C:GLU92:OE2	4.08761	Electrostatic	Attractive Charge	A:ARG41:NH2	Positive	C:GLU92:O E2	Negative
A:HIS431:ND1 - A:GLU34:OE2	2.73421	Hydrogen Bond	Conventional Hydrogen Bond	A:HIS431:ND1	H-Donor	A:GLU34:O E2	H-Acceptor
C:LYS122:N - A:SER19:OG	2.65273	Hydrogen Bond	Conventional Hydrogen Bond	C:LYS122:N	H-Donor	A:SER19:O G	H-Acceptor
A:SER19:OG - C:LYS122:O	2.17186	Hydrogen Bond	Conventional Hydrogen Bond	A:SER19:OG	H-Donor	C:LYS122: O	H-Acceptor
A:CYS28:SG - C:VAL93:O	2.68183	Hydrogen Bond	Conventional Hydrogen Bond	A:CYS28:SG	H-Donor	C:VAL93:O	H-Acceptor
A:LYS341:CE - A:TYR20:OH	3.34477	Hydrogen Bond	Carbon Hydrogen Bond	A:LYS341:CE	H-Donor	A:TYR20:O H	H-Acceptor
C:PRO88:CD - A:GLN44:OE1	3.07187	Hydrogen Bond	Carbon Hydrogen Bond	C:PRO88:CD	H-Donor	A:GLN44:O E1	H-Acceptor
A:GLY46:CA - C:LYS125:O	2.77854	Hydrogen Bond	Carbon Hydrogen Bond	A:GLY46:CA	H-Donor	C:LYS125: O	H-Acceptor
A:ARG382 - A:PRO33	5.25705	Hydrophobic	Alkyl	A:ARG382	Alkyl	A:PRO33	Alkyl
C:ILE94 - A:MET26	4.51661	Hydrophobic	Alkyl	C:ILE94	Alkyl	A:MET26	Alkyl
C:CYS133 - A:PRO24	5.01849	Hydrophobic	Alkyl	C:CYS133	Alkyl	A:PRO24	Alkyl
A:PRO27 - C:ILE117	5.16089	Hydrophobic	Alkyl	A:PRO27	Alkyl	C:ILE117	Alkyl
A:CYS28 - C:ILE94	4.24299	Hydrophobic	Alkyl	A:CYS28	Alkyl	C:ILE94	Alkyl
A:PHE408 - A:PRO33	4.66588	Hydrophobic	Pi-Alkyl	A:PHE408	Pi-Orbitals	A:PRO33	Alkyl
C:PHE151 - A:VAL25	4.90491	Hydrophobic	Pi-Alkyl	C:PHE151	Pi-Orbitals	A:VAL25	Alkyl
A:TYR20 - A:LYS362	5.17424	Hydrophobic	Pi-Alkyl	A:TYR20	Pi-Orbitals	A:LYS362	Alkyl
A:HIS45 - C:ILE80	5.097	Hydrophobic	Pi-Alkyl	A:HIS45	Pi-Orbitals	C:ILE80	Alkyl
A:HIS45 - C:LEU87	5.19308	Hydrophobic	Pi-Alkyl	A:HIS45	Pi-Orbitals	C:LEU87	Alkyl
**Apisimin (Q86BU7)**							

C:ASN54:ND2 - C:GLN21:OE1	2.88757	Hydrogen Bond	Conventional Hydrogen Bond	C:ASN54:ND2	H-Donor	C:GLN21:OE1	H-Acceptor
**Hymenoptaecin (C7AHW3)**							
C:LYS72:NZ - A:GLY80:OXT	4.28561	Electrostatic	Attractive Charge	C:LYS72:NZ	Positive	A:GLY80:OXT	Negative
C:ARG96:NH2 - A:ASP62:OD2	4.94142	Electrostatic	Attractive Charge	C:ARG96:NH2	Positive	A:ASP62:OD2	Negative
A:SER360:OG - A:ASP60:OD1	2.88055	Hydrogen Bond	Conventional Hydrogen Bond	A:SER360:OG	H-Donor	A:ASP60:OD1	H-Acceptor
A:ARG382:NH1 - A:GLN51:O	3.28408	Hydrogen Bond	Conventional Hydrogen Bond	A:ARG382:NH1	H-Donor	A:GLN51:O	H-Acceptor
A:ARG382:NH2 - A:GLN51:O	3.01298	Hydrogen Bond	Conventional Hydrogen Bond	A:ARG382:NH2	H-Donor	A:GLN51:O	H-Acceptor
A:ARG382:NH2 - A:VAL52:O	3.02735	Hydrogen Bond	Conventional Hydrogen Bond	A:ARG382:NH2	H-Donor	A:VAL52:O	H-Acceptor
A:ARG382:NH2 - A:TYR68:OH	2.61612	Hydrogen Bond	Conventional Hydrogen Bond	A:ARG382:NH2	H-Donor	A:TYR68:OH	H-Acceptor
A:TYR403:OH - A:THR50:OG1	3.28194	Hydrogen Bond	Conventional Hydrogen Bond	A:TYR403:OH	H-Donor	A:THR50:OG1	H-Acceptor
A:LYS477:NZ - A:THR46:O	3.19022	Hydrogen Bond	Conventional Hydrogen Bond	A:LYS477:NZ	H-Donor	A:THR46:O	H-Acceptor
A:GLN51:NE2 - A:ASP428:OD2	3.06886	Hydrogen Bond	Conventional Hydrogen Bond	A:GLN51:NE2	H-Donor	A:ASP428:OD2	H-Acceptor
A:TYR63:OH - C:GLU143:OE1	2.45855	Hydrogen Bond	Conventional Hydrogen Bond	A:TYR63:OH	H-Donor	C:GLU143:OE1	H-Acceptor
C:ARG96:CD - A:ASP60:OD2	3.72574	Hydrogen Bond	Carbon Hydrogen Bond	C:ARG96:CD	H-Donor	A:ASP60:OD2	H-Acceptor
A:TYR68:OH - A:PHE408	3.30272	Hydrogen Bond	Pi-Donor Hydrogen Bond	A:TYR68:OH	H-Donor	A:PHE408	Pi-Orbitals
A:ARG382 - A:ARG54	4.82596	Hydrophobic	Alkyl	A:ARG382	Alkyl	A:ARG54	Alkyl
C:PRO88 - A:MET73	4.51477	Hydrophobic	Alkyl	C:PRO88	Alkyl	A:MET73	Alkyl
C:LYS91 - A:ILE61	4.41457	Hydrophobic	Alkyl	C:LYS91	Alkyl	A:ILE61	Alkyl
C:VAL93 - A:ILE61	4.43001	Hydrophobic	Alkyl	C:VAL93	Alkyl	A:ILE61	Alkyl

**Melittin (I3RJI9A)**							
A:LYS402:NZ - C:GLU24:OE1	4.92696	Electrostatic	Attractive Charge	A:LYS402:NZ	Positive	C:GLU24:OE1	Negative
A:SER472:OG - C:GLU26:OE1	3.27308	Hydrogen Bond	Conventional Hydrogen Bond	A:SER472:OG	H-Donor	C:GLU26:OE1	H-Acceptor
A:SER472:OG - C:GLU26:OE2	3.14113	Hydrogen Bond	Conventional Hydrogen Bond	A:SER472:OG	H-Donor	C:GLU26:OE2	H-Acceptor
C:GLU30:OE2 - A:TYR451	2.70622	Electrostatic	Pi-Anion	C:GLU30:OE2	Negative	A:TYR451	Pi-Orbitals
C:ALA28 - A:ILE450	3.35757	Hydrophobic	Alkyl	C:ALA28	Alkyl	A:ILE450	Alkyl

Figure S1. Homology modeled 3D structure of selected BAMPs along with Ramachandran plot analysis, and PROSA validation showing Z-score. (A) Abaecin, (B) Apamin, (C) Apisimin, (D) Hymenoptaecin, (E) Melittin.

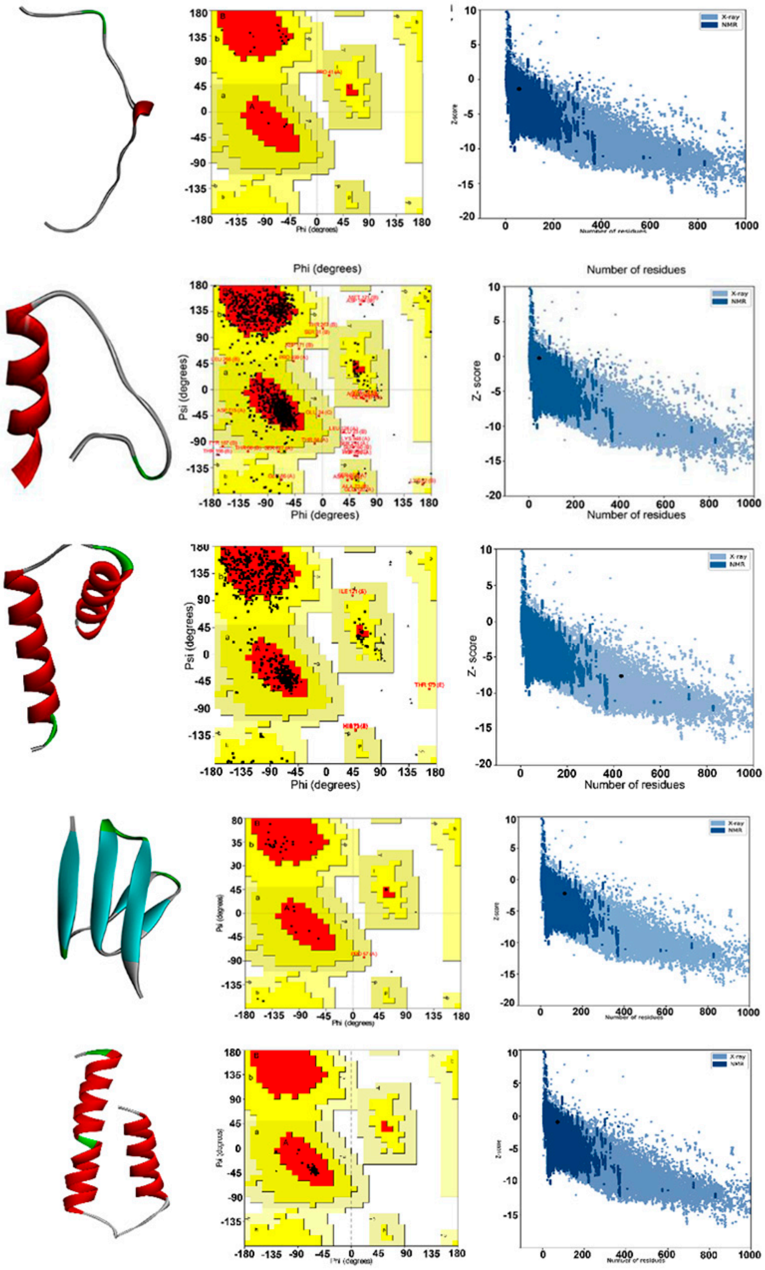

Figure S2. Three-dimensional structure of the human toll-like receptor with its PDB accession ID. (A) Human toll-like receptor 3 (PDB ID: 1ZIW), B. Human Toll-like receptor 4-MD2 complex (PDBID:3FXI).

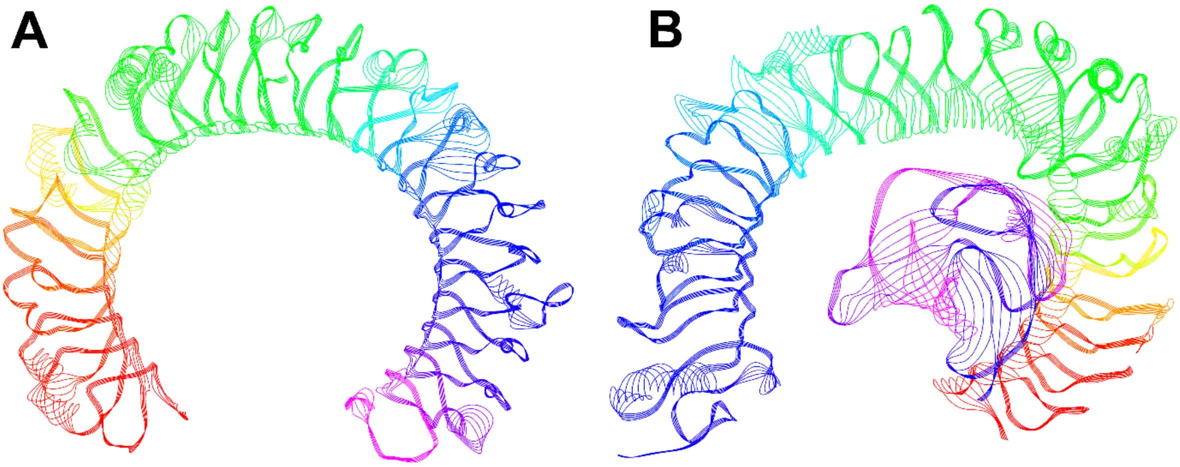

Tertiary structure of human TLR3 (PDBID: 1ZIW)

Tertiary structure of human TLR4-MD2 complex (PDBID: 3FXI)

Figure S3. Three-dimensional view of the movement of top hit receptor–ligand complexes for BAMPs and TLR3, every 20 ns during the period of 0-100 ns of molecular dynamics simulations. (A) Abaecin (B) Apamin (C) Apisimin (D) Hymenoptaecin (E) Melittin.

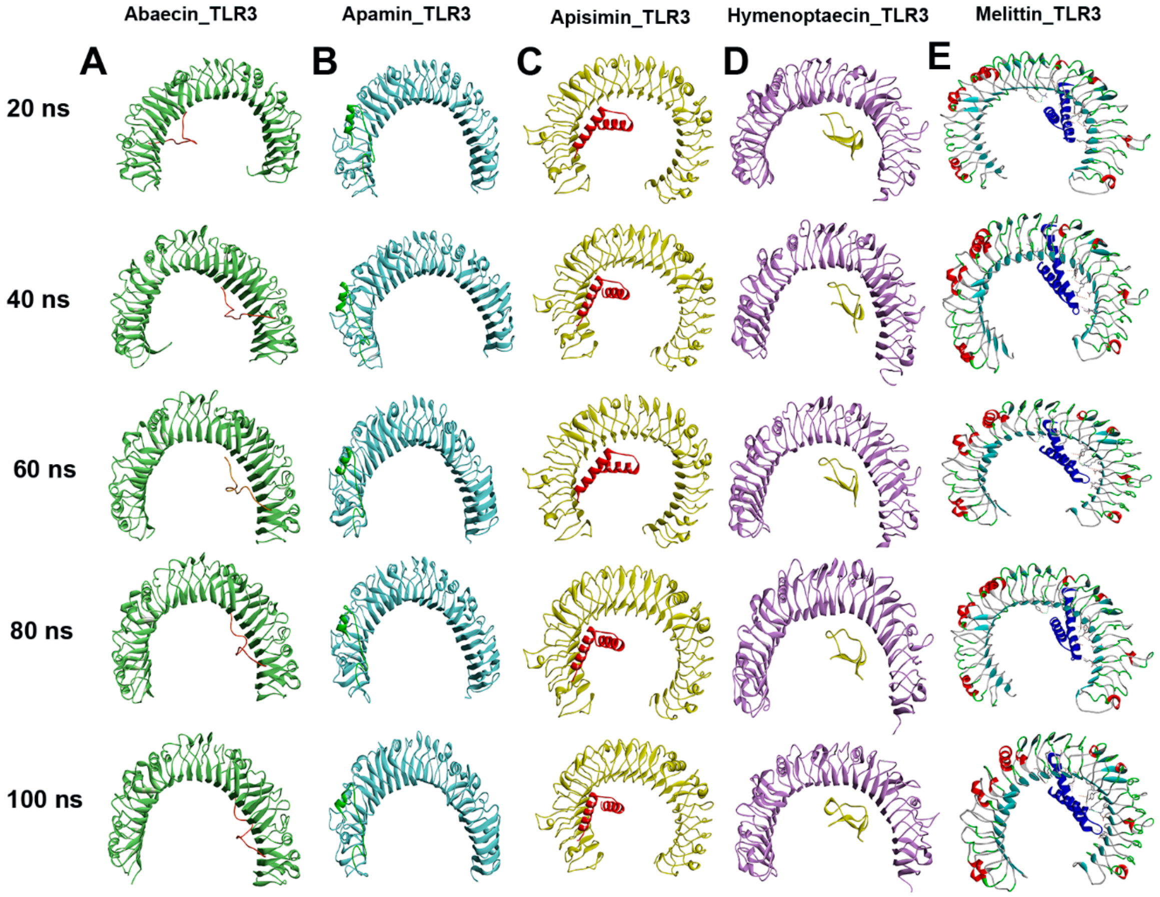

Figure S4. Three-dimensional view of the movement of top hit receptor–ligand complexes for BAMPs and TLR4-MD2, every 20 ns during the period of 0-100 ns of molecular dynamics simulations. (A) Abaecin (B) Apamin (C) Apisimin (D) Hymenoptaecin (E) Melittin.

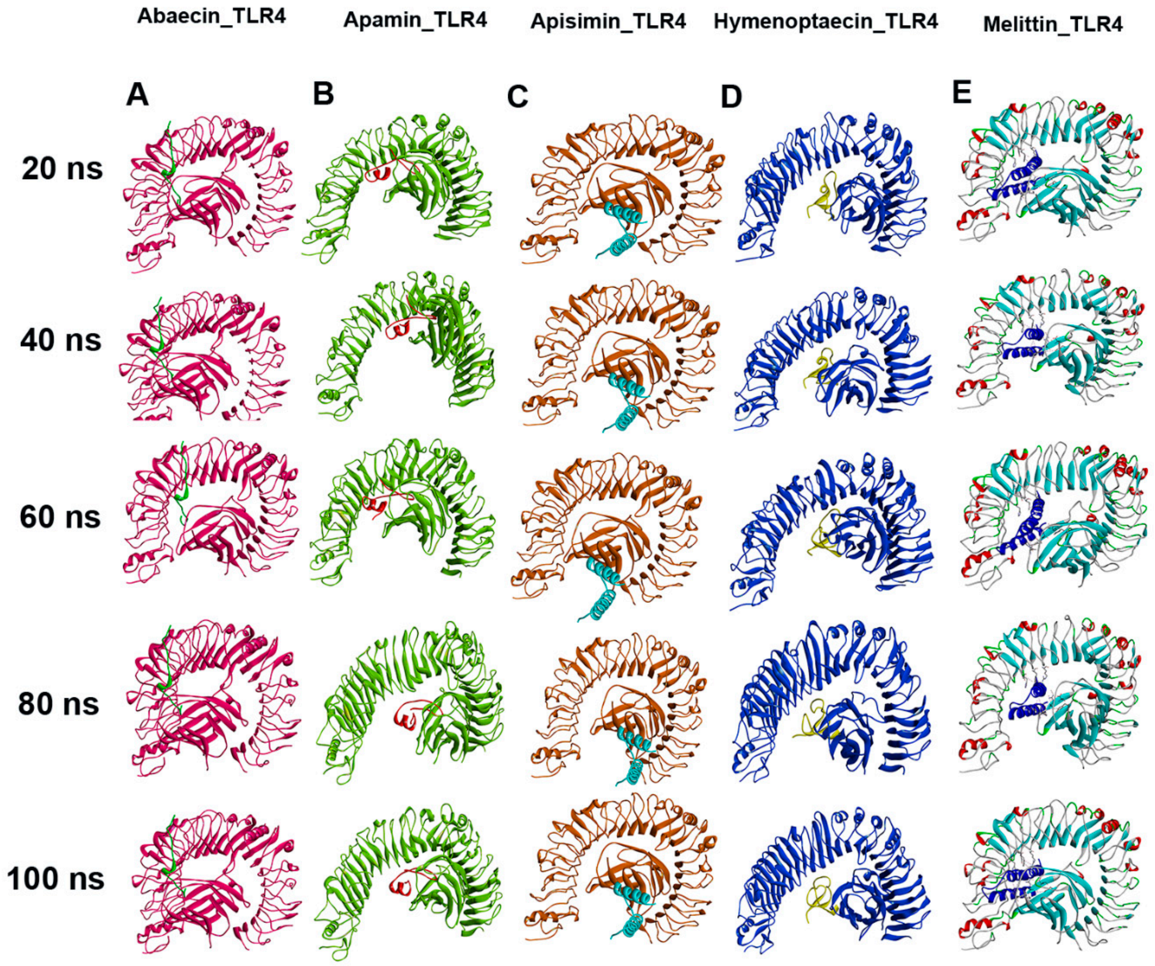

Supplement: Supplementary file 1 [file cimb-48-00081-s001.zip › cimb-4051748-supplementary.pdf]
